# Multi‐View Biomedical Foundation Models for Molecule‐Target and Property Prediction

**DOI:** 10.1002/advs.202517840

**Published:** 2026-01-28

**Authors:** Parthasarathy Suryanarayanan, Yunguang Qiu, Shreyans Sethi, Diwakar Mahajan, Hongyang Li, Yuxin Yang, Elif Eyigoz, Aldo Guzmán‐Sáenz, Daniel E. Platt, Timothy H. Rumbell, Kenney Ng, Sanjoy Dey, Myson Burch, Bum Chul Kwon, Pablo Meyer, Feixiong Cheng, Jianying Hu, Joseph A. Morrone

**Affiliations:** ^1^ IBM TJ Watson Research Center 1101 Kitchawan Rd. Yorktown Heights NY USA; ^2^ Cleveland Clinic Genome Center, Lerner Research Institute Cleveland Clinic Cleveland OH USA; ^3^ Genomic Medicine Institute, Lerner Research Institute Cleveland Clinic Cleveland OH USA; ^4^ IBM Research ‐ Almaden 650 Harry Rd San Jose CA USA; ^5^ IBM Research 314 Main St Cambridge MA USA; ^6^ Department of Molecular Medicine Cleveland Clinic Lerner College of Medicine Case Western Reserve University Cleveland OH USA

**Keywords:** Alzheimer's disease, foundation models, molecular property prediction, virtual screening

## Abstract

Molecular foundation models hold promise to provide accurate predictions for a large and diverse set of downstream tasks in bio‐medical research. Quality molecular representations are key and foundation model development has typically focused on a single representation or molecular view, which may have strengths or weaknesses on a given task. We develop Multi‐view Molecular Embedding with Late Fusion (MMELON), an approach that integrates pre‐trained graph, image and text foundation models and may be readily extended to additional views and models. The multi‐view model performs robustly and is validated on over 120 tasks, including molecular solubility, ADME properties, and activity against G Protein‐Coupled receptors (GPCRs). The GPCR model array is leveraged to perform a virtual screen in search of ligands binding to Alzheimer's disease related GPCRs. We identify a number of such targets and employ the multi‐view model to select strong binders from a compound screen. Predictions are validated through structure‐based modeling and identification of key binding motifs.

## Graphical abstract

Molecular foundation models can provide accurate predictions for a large set of downstream tasks. We develop MMELON, an approach that integrates pre‐trained graph, image, and text foundation models and validate our multi‐view model on over 120 tasks, including GPCR binding. We perform a virtual screen for ligands that bind to Alzheimer's disease related GPCRs and validate through structure‐based modeling.

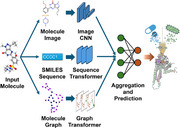

## 1 Introduction

Drug discovery is a complex, multi‐stage process. Lead identification and lead optimization remain costly with low success‐rates [1]. The prediction of a broad range of chemical and biological properties of candidate molecules is an essential component of screening and assessing molecules and data‐driven, machine learning approaches have long aided in this process [2, 3, 4, 5, 6].

Molecular representations form the basis of machine learning models [2, 7]. However, learning useful and generalized latent representation is a hard problem due to limited amounts of labeled data, wide ranges of downstream tasks, vast chemical space, and large heterogeneity in molecular structures. Learning latent representations using unsupervised techniques is vital for such models to scale. Foundation models, including large language models (LLMs) have revolutionized many fields [8] and similar sequence‐based foundation models have shown promise to learn “pre‐trained” molecular representations and be trainable on many downstream property prediction tasks [9, 10, 11, 12].

While sequence‐based representations have been successful in capturing human‐generated knowledge/information in LLMs, when it comes to representing the geometry of objects like molecules, it is likely that sequence molecular representations alone will not be sufficient. Geometry and composition give rise to chemical and biological properties [13]. Graph representations are naturally conducive to capturing molecular symmetries and other structural properties in a flexible manner [2, 14, 15, 16, 17]. However typical graph neural network (GNN) architectures lack the capacity to scale to large numbers of parameters and learn from very large datasets. Graph‐transformers can represent graphs in a high‐capacity neural network [18, 19] and are better candidates for foundation models. Image‐based representation paired with a convolutional neural network (CNN) architecture have been explored with promising results [20]. ImageMol, an exemplar of this approach, shows strong performance across many downstream tasks [21].

Many recent efforts have explored combining complementary molecular representations–including SMILES, fingerprints, molecular graphs, 2D images, and in some cases 3D conformers–to improve downstream property prediction [22, 23, 24, 25, 26, 27, 28, 29, 30, 31, 32, 33]. These methods vary considerably in which molecular views they incorporate, whether they include any large‐scale pretraining, how tightly coupled the fusion architecture is across modalities, and the breadth and realism of the benchmark suites used for evaluation. For example, a few models combine more than two representations: DLF‐MFF [29] fuses four modalities (fingerprints, 2D graphs, 3D spatial graphs, and images), and PremuNet [30] integrates 1D/2D/3D molecular views. However, these systems use relatively small encoders trained with purely supervised objectives on limited datasets, without the modularity or capacity of modern molecular foundation models. Contrastive or reconstruction‐based approaches such as CGIP [32] and MoleSG [27] pretrain multimodal architectures, but only for two views and always within a single jointly trained backbone and not via independently pretrained, plug‐and‐play encoders. Among existing systems, CGIP [32] is the only approach that leverages a large‐scale corpus (≈ 10M molecules). Most remaining methods rely on modest‐scale pretraining or exclusively supervised training, and evaluation typically covers a narrow subset of properties (e.g., 6–14 MoleculeNet or ADMET endpoints). Several approaches further require 3D conformers (e.g., PremuNet, DLF‐MFF), which are expensive to generate consistently across large libraries and may introduce dataset‐dependent artifacts.

Leveraging the suite of Biomedical Foundation Model (BMFM) technologies [34], we introduce MMELON, an approach that combines multiple foundation models within a late fusion architecture, to support the creation of multi‐view molecular models. Instead of training a single monolithic multimodal network, we compose multiple independently pretrained, foundation‐scale molecular encoders (graph, SMILES, image). This design enables plug‐and‐play upgrades of any individual encoder without retraining the entire stack, providing a flexible and forward‐compatible architecture not present in prior multi‐view models. We adopt a simple but expressive late‐fusion mechanism with learned gating weights, yielding interpretable contributions from each view. Unlike mid‐level or joint reconstruction/contrastive fusion, our method avoids brittle cross‐modal co‐training, minimizes modality interference, and scales reliably across heterogeneous encoders.

Whereas prior work typically evaluates on a limited set of MoleculeNet tasks, our multi‐view models are fine‐tuned on over 120 datasets encompassing a wide range of properties, including CYP inhibition and other ADME properties [35, 36, 37, 38]. Beyond curated benchmarks, we build models for GPCR activity. GPCRs are a family of membrane receptors that are frequently targeted in drug discovery campaigns. We extract from public sources a database of activity of small molecules with respect to over 100 GPCR targets. [39, 40]. This large dataset is used to fine‐tune over 100 models that are validated on a held out set. Code and models are available on GitHub (https://github.com/BiomedSciAI/biomed‐multi‐view) and Hugging Face (https://Huggingface.co/ibm‐research/biomed.sm.mv‐te‐84m).

MMELON‐based models are not simply validated, but put into practice. Our models are demonstrated in a virtual screen, applied to identity binders to Alzheimer's disease (AD)‐related GPCRs. AD is a progressive age‐related disorder that is anticipated to affect over 150 million people by 2050 [41]. Current AD drugs remain limited whereas two antibody‐based drugs that are recently approved by the FDA primarily target the early‐stage of AD [42]. This challenge is largely due to the lack of potential druggable AD targets beyond the well‐established targets, such as amyloid‐beta (Aβ) or apolipoprotein E (APOE) [43]. Emerging machine learning algorithms have greatly empowered target identification and small molecule discovery [3]. For example, NETTAG [44] provided key insights for treating AD by unraveling potential therapeutical targets, including G Protein‐Coupled receptors (GPCR) [45]. LISA‐CPI [46] identified promising small molecule treatments for pain by leveraging both target structure and ligand image information. Additionally, MRL‐Mol [47] offered a preliminary investigation on the enhancement of deep learning models by integrating different molecular data modalities. 33 GPCRs associated to AD are identified. We utilize MMELON models to screen two sets of compounds, gut metabolites and FDA approved drugs [48]. Strong binders are uncovered for AD‐related GPCRs ADA2A and FPR1. and predictions are validated by structure‐based in silico experiments that identify pharmacophores and binding modes.

This paper is organized as follows. We introduce the architecture and pre‐training protocol of the MMELON‐based foundation model in Section 2.1. Its flexibility and robustness is demonstrated, first on diverse set of benchmarks in Section 2.2, and next on large‐scale GPCR data in Section 2.3. It is applied to screen for binding molecules for AD‐related targets in 2.4. Discussion and conclusions are given in Section 3.

## 2 Results

### 2.1 Pre‐Trained Models and Data

**FIGURE 1:**
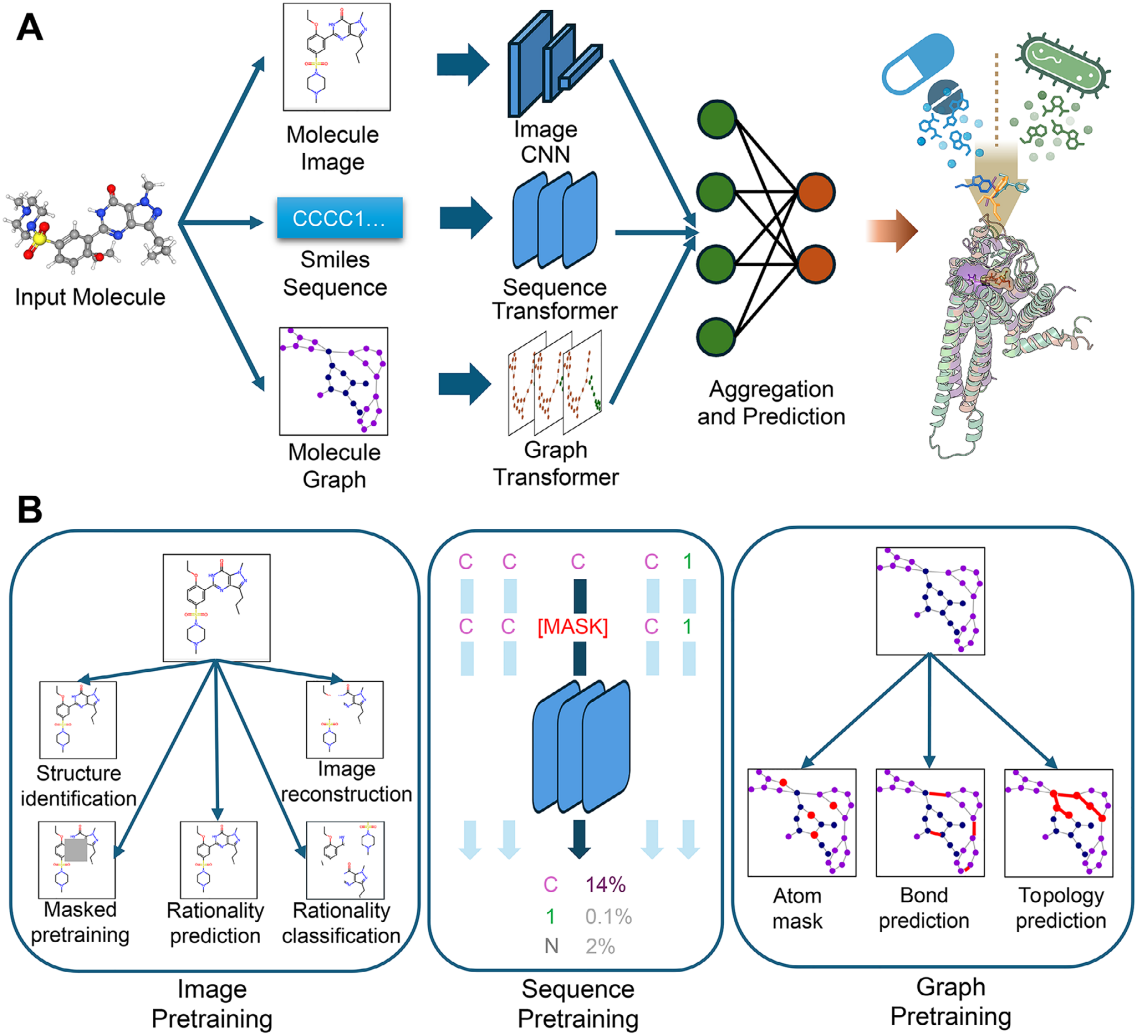
(A) Schematic of multi‐view architecture. Embeddings from three single view pre‐trained encoders (Image, Graph and Text) are combined by the aggregator module into a combined embedding. The network is fine‐tunable for downstream predictive tasks. (B) Pre‐training strategies for single view (Image, Text (sequence) and Graph) embeddings. These strategies are described in detail in Section 4.4.

We adopt three single view architectures and models based on image, text and graph molecular representations (Figure 1A). The models are herein referred to as Image, Graph, and Text, respectively. A schematic for the pre‐training strategies we employ is shown in Figure 1B. For Image, we adopt the ImageMol model [21]. ImageMol utilizes a CNN‐based architecture and is pre‐trained on 10M compounds selected from PubChem [49]. The pre‐trained model and weights are taken from ImageMol's publicly released checkpoint. This checkpoint is fine‐tuned using our independently developed code that is utilized here. For Text, our architecture and pre‐training strategy is adopted from MolFormer [12].

The Graph architecture is adopted from TokenGT [18]. This architecture processes chemically‐bonded graphs as a sequence of tokens before input to the transformer. We design three tasks to pre‐train the graph‐transformer: node feature masking, edge prediction, and Betti number prediction. Betti numbers are topological features that characterize loops and connected components in the molecular graph. They are useful to characterize more distant molecular connectivity. This work represents, to our knowledge, the first application of Betti numbers for pre‐training molecular foundation models. Further details of this and other pre‐training tasks are given in Section 4.4.

We pre‐train Graph and Text on a set of 200M molecules curated from two public data sources: PubChem and ZINC22 [49, 50]. The PubChem dataset contains a broad distribution of molecules including a minor population that is rather large and flexible, and as such of less interest for small molecule drug discovery. We filter out this minor population using a drug likeliness criteria [51]. An additional 120M molecules are randomly sampled from ZINC22. This set of molecules covers a distribution of drug like molecules and molecular properties (see Section 4.1 and Figures S1 and S2). Graph and Text are pre‐trained for 3 epochs each.

Similar to traditional cheminformatics descriptors, pre‐trained molecular embeddings capture chemical structure. We present a detailed analysis of the correlations in between the single‐view embeddings and cheminformatics descriptors in Appendix A and Figure S3. Analysis on the singe‐view embedding space indicates there is complementarity (measured by low‐to‐moderate correlation) between the chosen views, specifically between Image and Graph/Text, which may in turn be exploited by a multi‐modal fusion approach.

There are a number of multi‐modal fusion strategies that may be employed to combine representations. Table S1 summarizes representative multi‐view approaches and highlights differences in their input modalities, encoder architectures, fusion strategies, and evaluation scopes. We adopt a late fusion approach with interpretable learned gating because it readily integrates separately pre‐trained models in a ‘plug‐and‐play’ fashion and naturally supports analyses to gain insights into the role and importance of different modalities for different downstream tasks. In this approach, the singe‐views are first separately trained and then combined using an aggregator sub‐network (see Figure 1A). The single‐view foundation models serve as pre‐trained encoders that are utilized for downstream task fine‐tuning. We tested four different late fusion architectures and chose the one that overall performed the best over 8 fine‐tuning tasks (see Tables S2 and S3). Detailed descriptions of the late fusion aggregator and alternative architectures tested are given Section 4.5.

### 2.2 Multi‐View Foundation Model is Fine‐Tunable on Many Tasks

The prediction of electronic properties, ligand‐protein binding affinity, pharmokinetic properties, and toxicity are examples of tasks that are essential to chemistry and drug discovery. MoleculeNet [36] is a collection of datasets covering such tasks that serves as a common test for machine learning models. Performance on this suite of benchmarks is neither decisive, nor necessarily reflective of all important use‐cases in computational drug discovery, but currently it stands as one of the most widely used point of comparison between models. We fine‐tune MMELON on a selection of MoleculeNet tasks as well as a set of task for predicting the inhibition of cytochrome P‐450 (CYP) isoforms [35] which are critical in drug metabolism pathways, and the recently released ComputationalADME dataset, which include tasks such as liver microsomal stability and plasma protein binding [38] (see Table S4). In addition to the choice of dataset, the choice of data splitting is critical in robust assessment of model performance. We primarily adopt scaffold‐based methodologies, but also utilize random splits where appropriate. Section 4.8 for further discussion.

**FIGURE 2:**
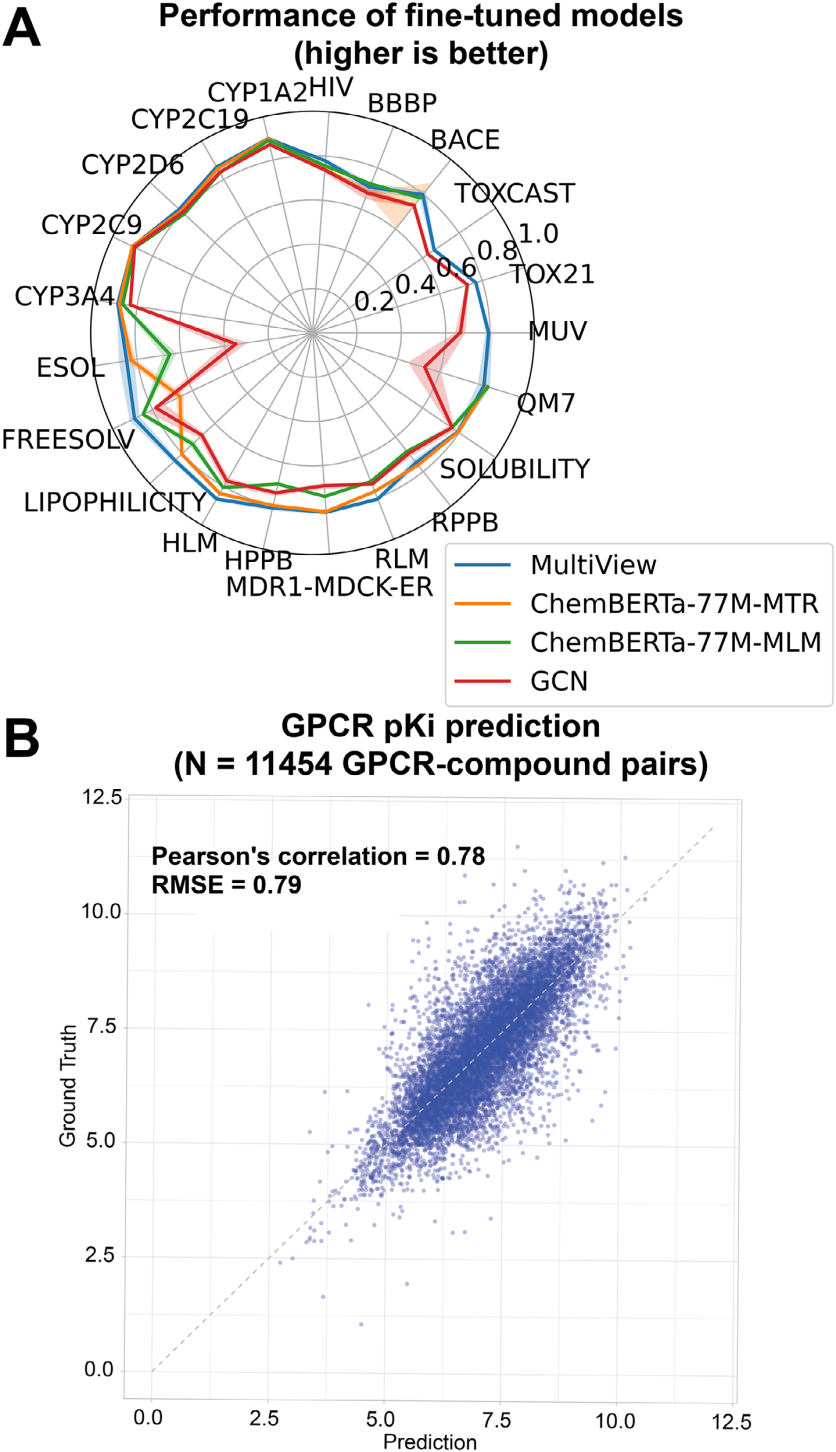
(A) Performance of the multi‐view model (blue line), ChemBERTa‐77M‐MTR (orange line), ChemBERTa‐77M‐MLM (green line), and GCN (red line) fine‐tuned on diverse downstream tasks. Train, validation, and test splitting is performed as described in the text. Classification tasks are characterized by ROC‐AUC. Regression metrics (RMSE, MAE) are scaled so that they can be plotted with ROC‐AUC values. The shaded region around the line denotes the 95% confidence interval based on fine‐tuning for each task with five trials initialized from different random seeds. (B) Correlation plot of predictions from models fine‐tuned on GPCR data versus experimental pKI values in the held out set. Values of the Pearson correlation and the RMSE are given in the legend.

Our multi‐view approach is first validated on MoleculeNet, CYP and ComputationalADME datasets. Scaled performance metrics for classification and regression tasks are reported in Figure 2A (see Section 4.8 for scaling procedure). Our model is compared against two models in the family of ChemBERTa text‐based molecular foundation models [9, 10], specifically ChemBERTa‐77M‐MLM and ChemBERTa‐77M‐MTR [10]. The former model is pre‐trained using a token masking strategy and the latter is pre‐trained on a panel of molecular property prediction tasks. To provide a simple baseline, results from a graph convolutional network (GCN) implemented with PyTorch Geometric [52] are also shown. More details of the fine‐tuning procedure are given in Section 4.8. The multi‐view foundation model clearly surpasses the GCN model, in agreement with previous foundation model based models, which are generally shown to outperform traditional ML or GNN‐based approaches [12, 21]. While ChemBERTa models are closer in performance to Multi‐View than the GCN, the Multi‐View model generally outperforms these models, specifically on a number of regression task (FREESOLV, LIPOPHILICTY, RLM, HLM) (see also Tables S5 and S8).

We perform ablation studies comparing the single‐view models to the multi‐view models on each task (Figure S4A and Tables S5–S8). We find that the multi‐view model performance is ‘robust’ in the sense that matches the performance of the top‐performing single‐view model across the broad spectrum of downstream tasks and does not produce a poor result on any tested dataset. Of the single view models, we find that overall, the Graph model outperforms Image and Text on our benchmark tests. The Graph model performs marginally worse than the other single views in only a handful of examples. The relative amount each view contributes to the fine‐tuned model are accessible in our multi‐view model (see Figure S4B). Consistent with the observation that Graph is the top‐performing single view, we find that graph is the largest contributor overall with some exceptions. See Appendix B and Figures S4–S6 for further discussion.

**FIGURE 3:**
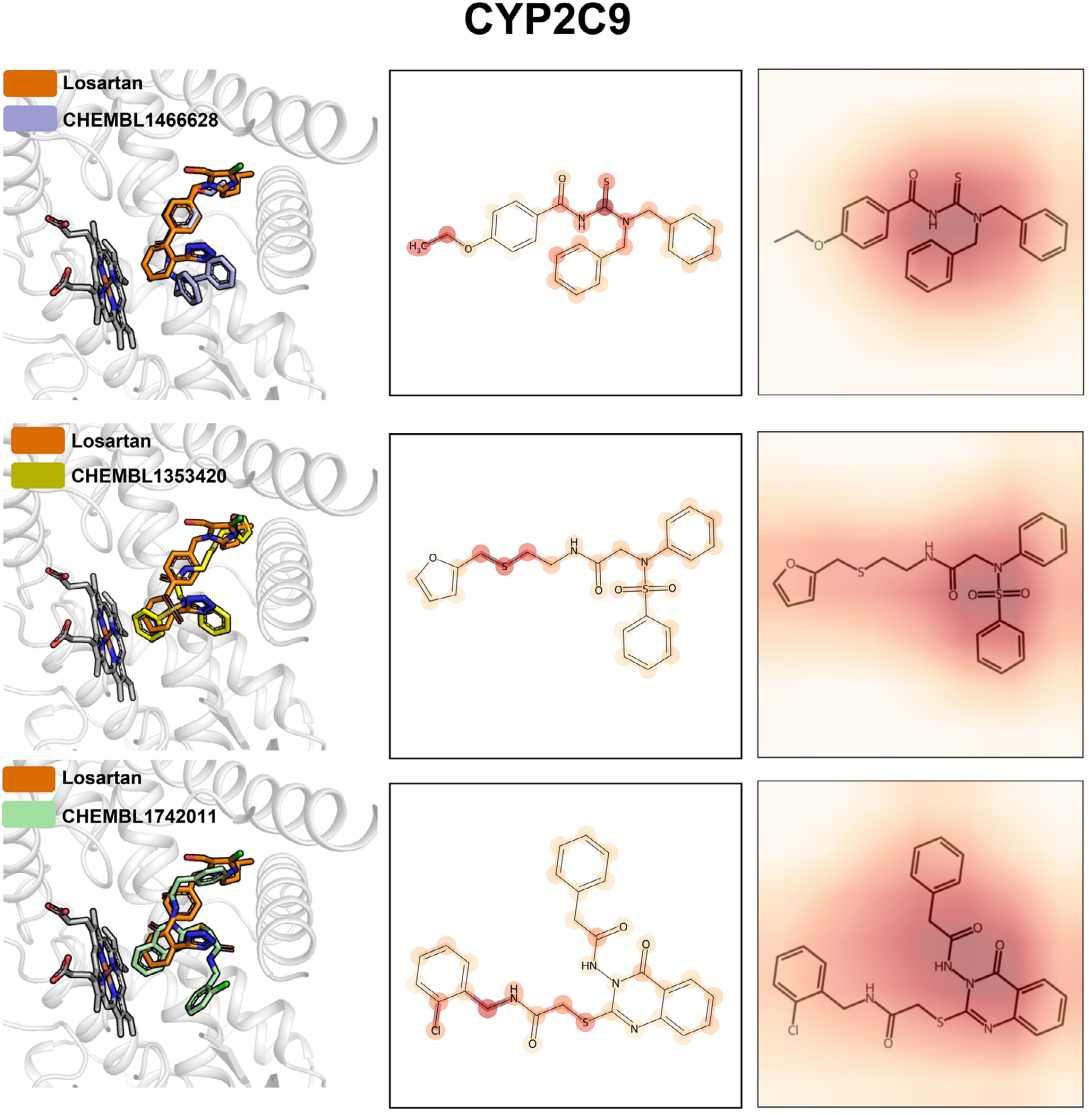
Docking poses of selected bioactive molecules against CYP2C9 as predicted by the multi‐view model are compared with the reported crystal structure (PDB: 8VX0, left column panels) and are shown alongside graph (center column panels) and image (right column panels) attention level heatmaps. Darker color indicates higher attention level. The heme group and remaining catalytic cite are rendered using the licorice and the ribbon style, respectively. ChEMBL IDs are given for selected molecules.

One set of tasks we highlight is cytochrome‐P450 (CYP) inhibition for five CYP isoforms [35]. Performance as measured by ROC‐AUC is high, ranging from 0.90 to 0.82 and results are comparable with SOTA performance [21]. For example, isoform CYP2C9 is one of most important contributor to drug metabolism [53] and our model achieved a high ROC‐AUC value (0.90, Figure 2A). The binding modes of top bio‐active molecules computed from molecular docking align well with reported structures of CYP2C9 in complex with the drug losartan [54] (PDB ID: 8VX0, see Figure 3). In addition, the salient molecular moieties as predicted by the graph and image views of multi‐view are both close to the catalytic site of CYP2C9 [54], suggesting our multi‐view models can highlight the important binding features.

In addition to tasks that take only small‐molecule information as input, we consider a task that combines our multi‐view embedding of small molecules with a popular sequence‐based protein embedding, ESM [55]. It is an open question what the protein embedding contributes to so‐called ‘drug‐target interaction’ tasks [16, 56, 57]. However, as ligand embeddings are known to drive model performance, this could still serve as another test of our small‐molecule models. We fine‐tuned our multi‐view and the single‐view models on the Davis dataset [37] describing the binding of 72 kinase inhibitors with 442 kinases. We find that the performance of our model exceeds that of a SOTA GNN implementation [57] (Figure S7).

### 2.3 Large‐Scale GPCR Models for Activity Prediction

GPCRs are important group of targets for drug discovery campaigns [58]. We leverage our base foundation model to build a resource that is utilized in virtual screening (see Section 2.4). We fine‐tune our multi‐view model on experimental binding affinity assays for 106 GPCR targets. This dataset contains experimental pKI values curated from ChEMBL [39] and GLASS [40]. All GPCRs datasets were retrieved but the GPCRs with fewer bioactivity measurements (<100) were not considered. Before training, the dataset is filtered to exclude molecules with a molecular weight >600 Da. This cutoff is commiserate with the molecular properties of the metabolites and drug molecules that are in the screening (inference) set. Datasets with less than 50 data points were removed from the set. Each assay/GPCR target is treated as a separate regression task and data is split using the balanced scaffold protocol [21].

**FIGURE 4:**
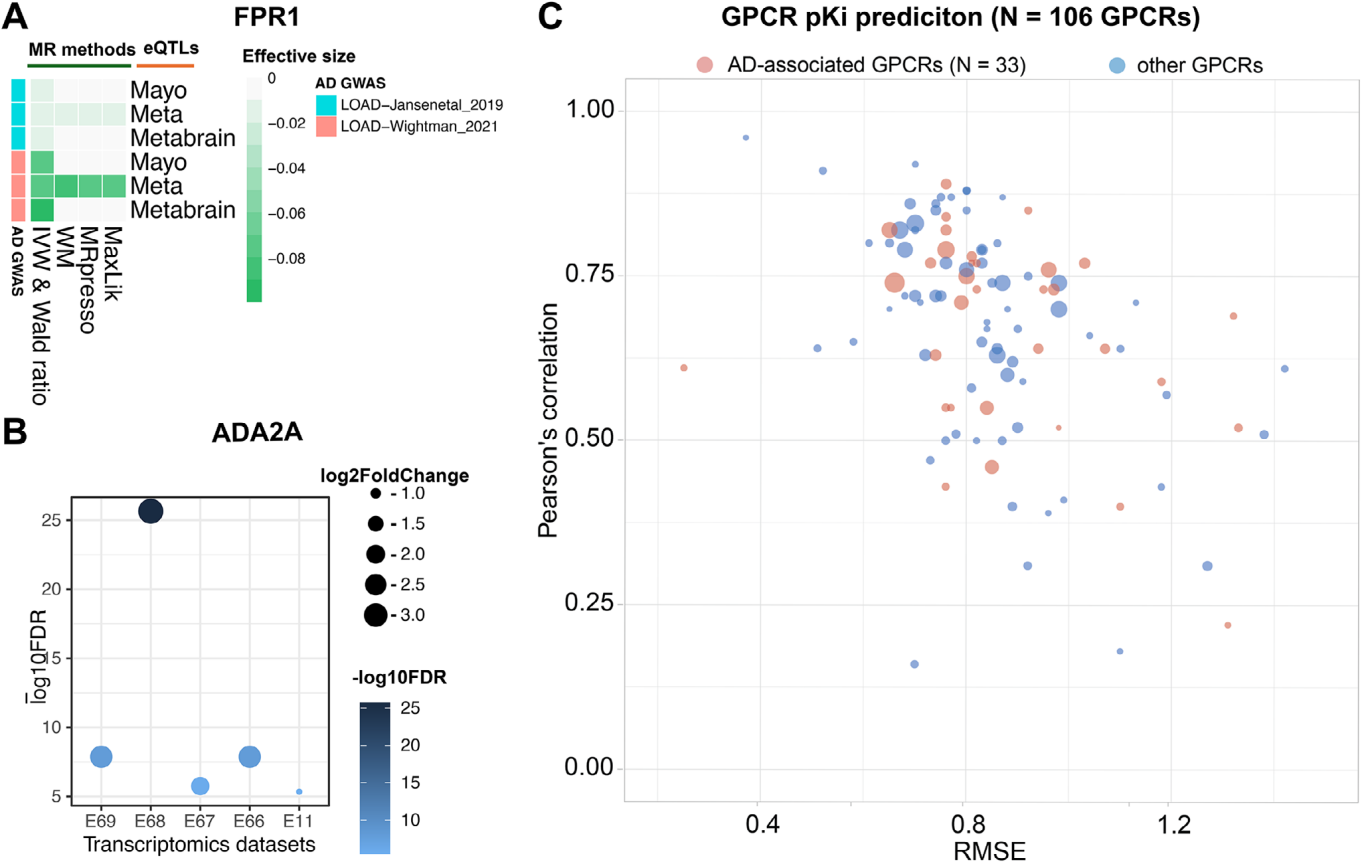
Genetics or multi‐omics evidenced AD‐related GPCRs FPR1 (A) and ADA2A (B). Genetics‐informed AD‐related FPR1 by Mendelian Randomization (MR). In total, three genomic datasets (Mayo, Meta, and Metabrain) in human cortex region were used. effective size <0 indicates elevated expression level of GPCRs decrease the likelihood of AD. Only data with FDR <0.05 was shown. Transcriptomics‐informed AD‐related ADA2A by investigating AlzGPS database (https://alzgps.lerner.ccf.org/). (C) Pearson correlation coefficient plotted against RMSE for 33 Alzheimer's disease related GPCR targets (red dots) and others (blue dots).

In Figure 2B, we plot the prediction on the held‐out set against the experimental values. The multi‐view fine‐tuned models are overall in good agreement with the held‐out set (Pearson correlation coefficient 0.78, average RMSE 0.79). To further validate our model, in Figure 4C we plot the Pearson correlation coefficient against the RMSE for each individual target. These metrics are negatively correlated, as expected. Over 80% of fine‐tuned models have RMSE<1 and nearly 70% have correlations >0.6 on the held‐out set.

### 2.4 Virtual Screening for AD‐Related GPCRs

A recent study by some of us identified a selection of GPCRs as potential drug targets for Alzheimer's disease [59]. In this study, we focus on uncovering AD‐related GPCRs within the set of targets we have fine‐tuned MMELON models. To unravel AD‐related GPCRs, genetics‐informed causal proteins inferred by Mendelian Randomization (MR) based on Genome‐Wide Association Studies (GWAS) [60, 61] and multi‐omics (transcriptomics and proteomics)‐informed differential expressed genes (DEGs) on the human brain [62] were inspected. Strong multi‐omics evidence‐supported protein was defined as those that were differential expressed in as least five datasets we previously compiled [62] (see Figure 4A,B and Table S9). The red dots in Figure 4C indicate the performance on the 33 AD‐related GPCRs is within range of the larger set (Pearson correlation coefficient: 0.67, average RMSE:0.88).

**FIGURE 5:**
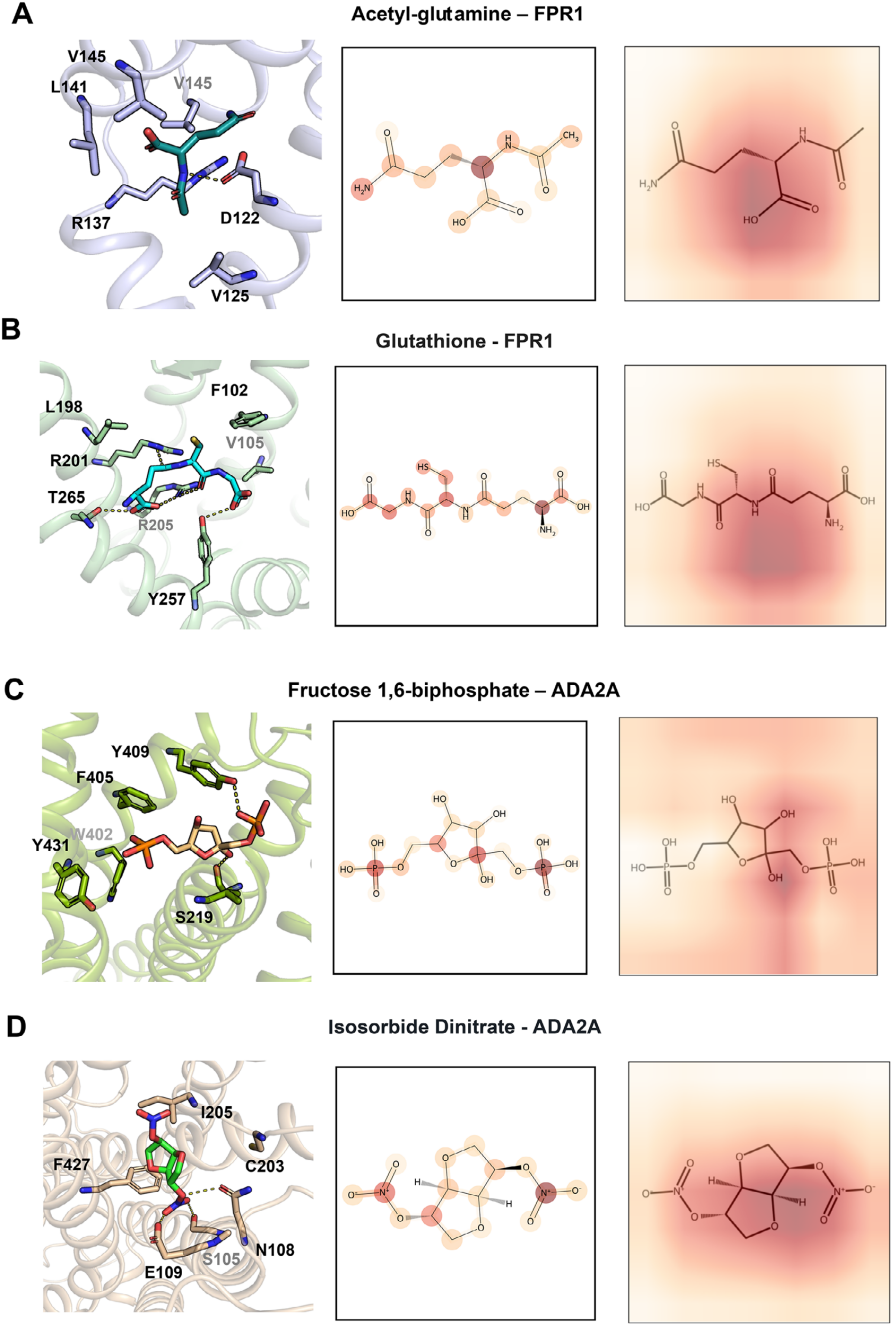
Examples of strong binders to FPR1 (A‐B) and ADA2A (C‐D) are depicted in the respective panels. Binding poses generated from docking software (left column panels) are depicted alongside heat maps on attention levels as predicted by the graph (center column panels) and image (right column panels) sub‐networks of our model. Darker color indicates higher attention level.

Next we sought to identify potential safe AD therapies from gut metabolites and FDA‐approved drugs. The validated multi‐view model is used to predict the binding affinity of a set of 515 gut metabolites [63] and 2,504 FDA‐approved drugs from DrugBank (Version 2021.1) [48]. We consider interactions of molecules in our virtual screen with specific AD‐related receptor. FPR1 is suggested as a genetics‐informed GPCR. High level of FPR1 is causally associated with increased risks of AD (Beta = ‐0.1, FDR = 0.001, Figure 4A). Through inspecting top ten small molecules in our screen, we found that a gut metabolite, acetyl‐glutamine is predicted to interact with FPR1 (multi‐view score: 6.8). By re‐analyzing the abundance of gut metabolites in microbial strains in vitro [63], acetyl‐glutamine was determined to have a high level in *Ruminococcus gnavus* (log2FoldChange: 1.19), a strain that mono‐colonized in mice performed better on a spatial working memory test [64]. Additionally, over‐expression of Aβ protein is significantly characterized by the decreased *Ruminococcus gnavus* [65]. 3D binding interaction analysis revealed that acetyl‐glutamine is located in an allosteric binding site that is distinct from the classical binding site, indicating a potential regulation mechanism (Figure 5A). For top predicted drugs, glutathione (GSH), an anti‐oxidant drug [66], GSH, is prioritized (multi‐view score: 6.53) to interact with FPR1. GSH is a nutrition supplementation that consist of three amino acids, including glutamic acid [67]. The depletion of GSH has been found in the hippocampal regions in mild cognitive impairment (MCI) and AD patients compared to healthy control [68]. GSH forms strong hydrogen bond interactions with FPR1 in the classic binding site (Figure 5B). We find that image (α=0.51) and graph (α=0.39) are the top contributors to the FPR1 multi‐view model and generate attention heat maps from the image and graph sub‐networks (Figure 5A,B). Most of the functional groups of acetyl‐glutamine (e.g., amide group) and GSH have high attentions. For acetyl‐glutamine binding to FPR1, the image highlights the attention of amide group that forms hydrogen bond with FPR1 while the graph captures the main chain carbon that controls the direction of amide group, which is consistent with the binding mode.

ADA2A is a GPCR with the strong multi‐omics evidence to have a significant lower expression in AD (differential expressed in 1 human and 4 mouse transcriptomics datasets, Figure 4B) [62]. A gut metabolite, fructose 1,6‐biphosphate, is predicted to strongly interact with ADA2A (multi‐view score 7.37). Fructose 1,6‐biphosphate is involved in glycolysis, the level of which was down regulated in APP/PS1 mice [69]. High levels of 1,6‐biphosphate have been detected in genus *Mitsuokella* (log2FoldChange: 13.42), which is decreased in Aβ+ (positive) patients [70]. Fructose 1,6‐biphosphate is predicted to bind a classic site of ADA2A (Figure 5C). Isosorbide dinitrate, a drug used to prevent and treat chest pain caused by coronary artery disease [48], is predicted as a top candidate to bind ADA2A (multi‐view score: 8.21, Figure 5D). The high attention of fructose 1,6‐biphosphate and isosorbide dinitrate were captured for key chemical moieties (Figure 5C,D). Although the image serves as the primary contributor (α=0.76), the graph representations play a crucial supplementary role (α=0.22) to preserve functional atoms, such as phosphorus atoms in phosphate of fructose 1,6‐biphosphate and nitrogen atoms in nitro groups of isosorbide dinitrate, all of which establish key interactions with ADA2A.

## 3 Discussion

A molecular foundation model that is fine‐tunable on a broad, diverse set of tasks can serve as a crucial component of any discovery workflow. The choice of molecular representation used in such a model is crucial. Each choice and individual representation (view) may have strengths and weaknesses and a model that performs robustly over a wide range of property prediction tasks is desired. Here, we introduce MMELON, a multi‐view architecture that combines molecular representations from multiple embeddings using an interpretable late fusion approach, utilizing foundation models based on three common representations of chemical structure, Graph, Image, and Text. We make code and models available on GitHub (https://github.com/BiomedSciAI/biomed‐multi‐view) and Hugging Face (https://Huggingface.co/ibm‐research/biomed.sm.mv‐te‐84m).

Overall, while existing multi‐view models provide valuable insights into combining molecular representations, none offer the modularity, scalability, or practical breadth of our architecture. Our work introduces a complementary direction in multimodal molecular foundation modeling by decoupling single‐view pretraining from downstream multi‐view fusion, enabling robust, interpretable, and application‐ready molecular prediction at scale. We believe this places our work as a distinct and complementary contribution in the landscape of molecular foundation modeling.

One area where molecular foundation models hold promise is that they can be readily applied to many downstream tasks. Therefore, applying foundation models beyond a set of standard benchmarks is vital to demonstrate their utility. We fine‐tune our multi‐view models on over 120 datasets that span diverse tasks including binding activity, drug metabolism, and ADME properties. This is, to our knowledge the largest number of datasets on which molecular foundation models have been applied to in the literature. The multi‐view models perform robustly across a wide range of property prediction tasks involving both classification and regression, matching ‐ and in some cases exceeding ‐ the top‐performing single‐view model on that specific dataset and performing well when compared with ChemBERTa pre‐trained models that are fine‐tuned on our data [10]. Rigorous quantitative comparison with this and other models, including other multi‐view models, requires fine‐tuning on the same data splits and the availability of code to make this possible. Future work in the field should focus on developing platforms that facilitate such comparisons.

In this study, we specifically focus on the curation of a large collection of GPCR assays which we use to fine‐tune over 100 activity models. Model performance is validated against held‐out sets, and the array of GPCR models can serve as a panel for large‐scale screens. To this end, we apply MMELON to a virtual screen of Alzheimer's disease related GPCRs. We first identify a number of GPCR targets that are implicated in Alzheimer's disease. Then several strong binders to these targets are identified from the metabolite and drug datasets. They are studied by molecular docking [59] and pharmocophore identification, showing promising results. Future work will aim to further validate these predictions through wet‐lab experiments.

All views that are currently integrated into the MMELON architecture are ultimately derived from so‐called 1D (string) or 2D (graph and image) representations [71]. More expressive representations including 3D dimensional conformers [72, 73], and molecules in the context of binding [16, 74] can be readily incorporated into MMELON. Combining insights from the molecular docking that is used to validate our approach [59] is also a potential next step. Future work will also further explore alternative training and multi‐modal fusion strategies. Besides molecule‐target and property prediction, this approach can be leveraged for molecule generation and lead optimization as well. Furthermore, while the small molecule use case is explored here, the approach itself may be readily extended to include proteins and other macromolecules.

## 4 Experimental Section

### 4.1 Pre‐Training Data

The objective of the pre‐training dataset was to develop a task‐agnostic representation through self‐supervision. Choosing a dataset with the right diversity and relevance to downstream tasks leads to compute‐optimal richer representations. We use two main sources of molecular data. (1) PubChem [49] is a public chemical database managed by the National Center for Biotechnology Information (NCBI). It aggregates data on small molecules from over 870 sources, including information on substances, bioassays, protein targets, genes, pathways, cell lines, taxonomy, and patents. (2) ZINC22 [50] is an ultra‐large database containing 37 billion molecules, focusing on those with fewer than 30 heavy atoms to ensure dense coverage of “drug‐like” chemical space.

Various studies [75] indicate that the required dataset size correlates with the number of parameters used, even though the scaling laws for molecular foundation models remain to be explored fully. Furthermore, training on larger datasets tends to yield diminishing returns. For example, MolFormer was trained using a combination of PubChem and ZINC databases, encompassing a total of 1.1 billion molecules. Notably, MolFormer trained on a smaller dataset (10% ZINC + 10% PubChem) performed nearly, as well as the model trained on a dataset ten times larger across various downstream tasks [12]. Combining only 10% of ZINC with 10% of PubChem did not show reduced performance compared to training solely on ZINC.

Our pre‐training dataset comprises a collection of molecules sourced from the PubChem [49] and ZINC22 [50] chemical databases. The current size of the dataset is 200 million molecules. From PubChem, a “drug‐like” subset of 80 million molecules was curated. The curation process involved extracting the largest fragment of each molecule, removing duplicates, and filtering based on the following drug‐likeness criteria:

- Molecular weight ≤600 Da
- ≤5 hydrogen bond donors
- ≤10 hydrogen bond acceptors
- ≤10 rotatable bonds

From the ZINC22 dataset, 120 million molecules were sampled. ZINC22 is an ultra‐large database containing 37 billion molecules, focusing on those with fewer than 30 heavy atoms to ensure dense coverage of chemical space containing “drug‐like” molecules. Previous studies had extensively utilized these datasets. For example, MolFormer used the complete PubChem database combined with one billion molecules from ZINC, while ImageMol utilized 10% of PubChem's data. Figures S1 and S2 illustrate the distribution of molecular properties of the pre‐training dataset.

### 4.2 Input Representation

#### 4.2.1 Graph Representation

The molecular graph representation encoded a molecule as an undirected graph G=(V,E), where V represents the set of atoms as nodes and E represents the set of bonds as edges between the nodes. We employed deep graph featurization from [76]. Each node v∈V was associated with a feature vector $h_{v}\in R^{d}$, which encoded atom‐specific properties such as atomic number, chirality, degree, formal charge, and hybridization state. Similarly, each edge $e=(v_{i},v_{j})\in E$, representing a bond between atoms $v_{i}$ and $v_{j}$, was characterized by a feature vector $h_{e}$, which includes bond‐specific properties such as bond type, bond direction, stereochemistry, conjugation, and whether the bond was part of a ring. The node and edge features were then embedded into 512 D vectors using categorical embedding techniques, with each feature mapped to an embedding space of dimension 128. Graph Laplacian was computed over the molecular graph G, yielding the eigenvalues and eigenvectors that describe the graph's structure. This additional spectral information was used as positional encoding similar to [18].

#### 4.2.2 Image Representation

The image representation captures the 2D visual depiction of the molecular structure, providing a spatially coherent view of atomic arrangements and functional groups. Images often highlight molecular features such as symmetry, bond angles, and ring structures, which were essential for understanding molecular shape and stereochemistry. The input representation for the image model in the framework follows the approach outlined in ImageMol but was reproduced here for completeness. The molecular images created using RDKit [77] were processed as fixed‐size pixel grids, where each pixel grid encodes local chemical environment information. This view allowed the model to focus on both global molecular topology and local substructure details. The images undergo several preprocessing steps to ensure consistency and optimize the learning process. During training, data augmentation techniques were applied to the molecular images, introducing random transformations such as cropping and flipping. This augmentation process increases the model's robustness by simulating variations in the data. For validation and testing, only central cropping is applied, standardizing the image size to 224×224 pixels without altering the original molecular structure. A normalization step follows, scaling the pixel values to a standardized range for efficient learning.

#### 4.2.3 Text Representation

The input representation for our Text model follows the approach outlined in MolFormer [12], which was a molecular transformer model that processes chemical structures represented as SMILES (Simplified Molecular Input Line Entry System) strings [78]. For clarity, we describe the process here. The SMILES strings were tokenized using a custom tokenizer built upon a predefined vocabulary. The tokenizer employed a regular expression to capture key molecular substructures, atoms, bonds, and other chemical tokens, such as atoms (e.g., C, O, N), bond types (e.g., =, #, ‐), and structural elements like rings and branches. The regular expression used was adapted from the approach presented in Ref. [79]. The tokenized SMILES strings were embedded into continuous 768 D vectors.

### 4.3 Architectures

#### 4.3.1 Graph Model

The Graph model in our framework was based on the TokenGT architecture, which treated nodes and edges in a molecular graph as independent tokens. TokenGT provided two positional encoding schemes: orthogonal random features (ORFs) and Laplacian eigenvectors, which capture graph structure. In our implementation, we use Laplacian eigenvectors to encode positional information, as they capture the structural relationships between atoms through the graph Laplacian. The remaining layers of the model used a standard transformer architecture without graph‐specific modifications. By combining explicit featurization of tokens with the self‐attention mechanism of the transformer, graph model captures both local molecular features and global structural relationships without having to relearn easily computable features at the token level.

#### 4.3.2 Image Model

The Image model was based on the ImageMol architecture, which leverages a ResNet‐18 [80] convolutional backbone with residual blocks for hierarchical feature extraction from molecular images. For further architectural details, please refer to Ref. [21].

#### 4.3.3 Text Model

The Text model was built on the MolFormer architecture, which employed a standard transformer stack to embed tokenized SMILES sequence. Rotary positional embeddings were used to encode positional information in the sequence and a linear attention mechanism [81, 82] was used within its transformer architecture. For further architectural details, we refer the reader to Ref. [12].

#### 4.3.4 Model Size

The Graph model had 26M parameters, the Image model 11M parameters and the Text model 44M parameters. Including the aggregator sub‐network, the Multi‐View model had 84M parameters. The Text model accounts for ≈52% of the number of weights of Multi‐View and was of similar parameter size to earlier text‐based transformer models such as MolFormer [12].

### 4.4 Pre‐Training Tasks

The Text model was pre‐trained using a masked language modeling (MLM) task similar to Molformer. During pre‐training, 15% of the tokens in the SMILES sequences were randomly masked, and the model was trained to predict the masked tokens based on their context. The pre‐training objective minimizes the cross‐entropy loss between the predicted tokens and the true masked tokens.

The Image model was pre‐trained using five self‐supervised pretext tasks specified by ImageMol. These tasks include: (1) Multi‐Granularity Clustering Classification, which assigned pseudolabels at different levels of granularity to each molecule based on its chemical structure computed using Morgan fingerprints; (2) Molecular Rationality Discrimination, where the model distinguishes between rational and irrational molecular images; (3) Jigsaw Puzzle Prediction, where the model learns spatial relationships by solving shuffled molecular images; (4) Mask‐based Contrastive Learning, which ensures that masked molecular images were consistent with their unmasked counterparts in the latent space; (5) Molecular Image Reconstruction, to enforce consistency between the image and its encoding.

The Graph model was pre‐trained using three tasks designed to learn meaningful representations of molecular graphs. The first task was Masked Feature Prediction, where a portion (85%) of the token (both atoms and bonds) features was masked, and the model was trained to predict these masked features based on the surrounding graph structure. This encouraged the model to capture the local atomic environment and chemical composition. The second task was corrupted edge prediction, where 15% of randomly selected edges had the source/edge indices changed to a different value within the graph. The model was trained to predict which edges were corrupted and which were original, based on the token features and the overall graph structure. This helps the model learn the local molecular connectivity and the chemical viability of the edges.

#### 4.4.1 Pre‐Training on Topological Context

The third, novel pre‐training task involved predicting topological invariants of the molecular graph, specifically the Betti numbers, which were derived from simplicial homology. Betti numbers were used to describe the topological properties of a graph: $\beta_{0}$ represents the number of connected components, while $\beta_{1}$ captures the number of independent cycles within the graph. For each node $v_{a}$, the model predicts these Betti numbers for the subgraph around that node. For example, in Figure S8 at node $v_{a}$, the graph might had $\beta_{0}\left(S_{v_{a}}\right)=1$ (indicating a single connected component) and $\beta_{1}\left(S_{v_{a}}\right)=1$ (indicating the presence of one cycle). In contrast, at node $v_{b}$, the graph might exhibit $\beta_{0}\left(S_{v_{b}}\right)=2$ (two connected components) and $\beta_{1}\left(S_{v_{b}}\right)=0$ (no cycles). These Betti numbers provide both local and global structural information about the molecular graph, allowing the model to capture wider topological context at atom level representations.

### 4.5 Late Fusion Strategies

#### 4.5.1 Multi‐View Late Fusion Approach

We utilize a late‐fusion approach to combine Graph, Image, and Text Views. It was an attention‐based approach, inspired by Ref. [24] where the coefficients combining the $m^{\text{th}}$ view, $\alpha^{m}$, are the soft max outputs from an operation on embeddings $z^{m}$ performed on batch B (Equation (2)),

(1) $$\begin{array}{cc} z_{i}^{\text{mv}} & =\text{MLP}\left[\sum_{m\in M}\alpha^{m}z_{i}^{m}\right] \end{array}$$

(2) $$\begin{array}{cc} \alpha^{m}=\text{softmax}\left(w^{m}\right) & ,\;\;w^{m}=\frac{1}{B}\sum_{i\in B}q^{T}\cdot \tanh\left(W\frac{z_{i}^{m}}{\left|\right|z_{i}^{m}{\left|\right|}^{2}}+b\right)\; \end{array}$$

Before recovering the multi‐view embedding, $z^{\text{mv}}$, a multi‐layer perceptron is applied.

Equations (1) and (2) form the aggregator network of the multi‐view model. This network was subject to a secondary pre‐training. Aggregator pre‐training yields an initialization of the weights that was found to improve robustness during fine‐tuning and downstream task prediction. The aggregator was pre‐trained using an embedding reconstruction task on a set of 10M molecules randomly sampled from the dataset from which we pre‐train the Graph and Text models. At the conclusion of this secondary pre‐training, it was found that the Image, Graph and Text were weighted in decreasing order, with $\alpha_{\text{pret}}$ values of 0.6, 0.3, and 0.1, respectively. These values did not reflect singe‐view embedding quality or their contributions to specific down‐stream asks after subsequent fine‐tuning. Instead, the pre‐training task was related to difficulty by which a specific view could be reproduced (reconstructed) from the combined embedding and was consistent with the observation that Image was less correlated to the Graph and Text views (see Appendix A).

#### 4.5.2 Alternative Late Fusion Strategies

Before choosing our multi‐view model architecture, we explored several other late fusion strategies that combine the representations from the Graph, Text, and Image models. These strategies differ in how the inputs were prepared for the gating network. The gating network computed weights for each view, and the final output was a weighted aggregation of the view‐specific representations.

1) Projected Gating: The representations from each model were projected into a lower‐dimensional space. Let $d_{i}$ denote the dimensionality of the output from modality i, where i∈{1,2,3} corresponds to the graph, text, and image models. The total input dimension to the gating network is:

$$D_{\text{projected}}=\min\left(d_{1},d_{2},d_{3}\right)\times 3$$

The concatenated projected embeddings were passed into the gating network, which computes the weights $w_{i}$ for each modality.

2) Unprojected Gating: In this scheme, the original (unprojected) representations from the models were concatenated and used as input to the gating network. The input dimension in this case was the sum of the dimensions of the individual outputs:

$$D_{\text{unprojected}}=d_{1}+d_{2}+d_{3}$$

The gating network then computed the weights based on this concatenated unprojected representation. In both the schemes above, we computed weighted concatenation of the model output.

$$z_{\text{final}}=w_{1}z_{1}⊕w_{2}z_{2}⊕w_{3}z_{3}$$

3. Projected Gating With Feature Addition: This scheme the model outputs were first projected and concatenated, and the gating network computed the weights from this combined representation. The same aggregation process follows, where weights $w_{i}$ were applied to the corresponding expert outputs.

In this scheme, the final representation $z_{\text{final}}$ is computed as a weighted sum:

$$z_{\text{final}}=\sum_{i=1}^{3}w_{i}z_{i}$$

where $w_{i}$ are the modality‐specific weights and $z_{i}$ are the corresponding representations. Additionally, for interpretability, the average weights across the batch can be computed:

$${\bar{w}}_{i}=\frac{1}{N}\sum_{n=1}^{N}w_{i}^{\left(n\right)}$$

where N is the batch size.

The performance on a subset of MoleculeNet tasks for alternate schemes 1, 2, and 3 was compared against that of the multi‐view model in Tables S2 and S3. Overall, the chosen multi‐view architecture is the top performer.

### 4.6 Drug‐Target Interaction Model

Ref. [57] proposed a sequence‐based framework to investigate the binding affinities of proteins and ligands whilst experimenting with various embedding types for this purpose. From this study, we adapted their model for representing the protein structure that used convolutional neural network‐based encodings obtained from ESM‐1b [55]. We combined these CNN‐based protein embeddings with ligand embeddings obtained from our models. The combined embeddings were then passed through a prediction head, which was based on the prediction head of Ref. [57]. We used 64‐D protein and ligand encodings which were then concatenated to form a 128‐D embedding. The authors of Ref. [57] used double this size. We experimented with combining the protein embeddings with ligand embeddings obtained from various modalities of representation developed here: Image, Text, Graph, and multi‐view.

During training, the protein, single‐, and multi‐ view model weights were unfrozen gradually using the Xavier method. We used a batch size of 64 and a learning rate of 0.0005 for all models except for the Text model, for which we used a batch size of 128 and a learning rate of 0.00001. We trained for 2000 epochs, as suggested in Ref [57], and used the best performing model on the validation set to predict the test set. We used sixfold cross‐validation using one‐sixth of the data for validation, one‐sixth for testing, and the rest for training. The results were then averaged across the six cross‐validation folds.

### 4.7 Analysis of Select Classification Models

We consider the fine‐tuned output of the three single‐view and multi‐view models in single‐task classification and regression tasks and aim to perform comparative analyzes to uncover distinct characterizations of the models' predictions. For classification tasks, binarization was performed on outputs by applying a threshold of 0.5 to the sigmoid mapped scores. F1 scores were employed to evaluate the agreement between models. F1 scores compare binarized predictions typical of classifications, measuring how well the second model's predicted activities predict the first model's predicted activities. Note that this measure was not symmetric. For regression tasks, the model outputs for continuous prediction results were compared using the Pearson correlation coefficient, with a $\chi^{2}$ p‐value estimator.

### 4.8 Fine‐Tuning Protocols

The choice of data splitting into training, validation and test sets greatly impacts model performance. For example, The size‐ordered scaffold split clusters molecules according to their Murcko scaffold [83] and assigns the clusters to splits in size‐order. This was to be compared with other variations of scaffold split, which cluster the dataset in the same manner but assign clusters to train, validation, and test partitions randomly or ‘balanced’ (see, e.g. Ref. [21]). The balanced scaffold split assigned clusters large relative to the test set size to the training and other clusters randomly. Scaffold splits were generally understood to be more appropriate and harder than random splits, though the latter were still often utilized in benchmarks [36]. Following Ref. [21] size‐ordered scaffold and were used for MoleculeNet and balanced scaffold splits for CYP and GPCR tasks with an 0.8/0.1/0.1 train/validation/test splitting. For the ComputationalADME datasets it was noted that scaffold clustering yields a large number of singletons [38] so a random split (0.7/0.1/0.2 train/validation/test) was employed.

Hyper‐parameter optimization of the fine‐tuning process was important to ensure quality outputs. Optimization was first explored using Ray‐Tune [84]. Based on an exploration of our multi‐view model across a subset of MoleculeNet, a set of parameters for AdamW and regularization (e.g. weight‐decay) were chosen. Linear and MLP prediction heads were also explored. The learning rate was then varied for individual MoleculeNet tasks for Image, Graph, Text in the range of 1e‐5 to 1e‐3). In the case of the multi‐view model, the presence of multiple sub‐networks may call for a multiple optimizer fine‐tuning strategy that employed up to four learning rates in the aforementioned range. For CYP, ComputationalADME, and GPCR models, a learning rate was chosen across each set of tasks. The highest average validation performer across five seeds was chosen as the model instance to report the held‐out (test) set results.

To provide comparison with molecular foundation models in the literature, we fine‐tuned two ChemBERTa models, ChemBERTa‐77M‐MTR and ChemBERTa‐77M‐MLM [10] using the associated open source fine‐tuning script [85]. This script allowed for fine‐tuning on custom data splits. We fine‐tuned on the exact data splits used for our model evaluations. This assures a fair comparison, as results reported in the literature could vary widely. We modified the open source fine‐tuning to train for up to 100 epochs with an early stopping patience of ten epochs. Learning rate, seed, and batch size were varied during hyperparameter optimization [10]. Other settings were left ‘as‐is.’ As the ChemBERTa fine‐tune script did not appear readily compatible with multi‐task datasets, we omit MUV, TOX21, and TOXCAST from our comparison.

In order to facilitate the plots in Figure 2 and Figure S4, we scale the regression metrics according to the following formula $m^{\prime }=1-\left(m-m_{0}\right)/s$ where m is the unscaled metric and $m_{0}$ and s are an offset and scaling factor, respectively. For ESOL, FreeSolv, Lipophilicity, and QM7 the parameters $\left(m_{0},s\right)$ were (0.7,1.0), (1.5,5.0), (0.50,1.0), and (55.0,100.0), respectively. For the six ComputationalADME datasets, parameters (0.30,1.0) were used.

### 4.9 Genetics and Multi‐Omics Analysis

The genetics‐informed GPCRs prioritized by Mendelian randomization (MR) were retrieved from our previous study [59]. Briefly, 408 GPCRs were tested and publicly available four cis‐eQTL (Expression quantitative trait locus) datasets of human cortex region and three publicly available AD GWAS summary statistic datasets were used to test the causal effects of GPCRs to AD. Multi‐omics data of 88 bulk and single cell RNA‐seq transcriptome or proteome datasets were used, we previously compiled (available at AlzGPS: https://alzgps.lerner.ccf.org/) [62]. Briefly, differential expression analysis was performed on microarray, bulk RNA‐seq, and single‐cell/nucleus (sc/sn) RNA‐seq datasets. The threshold of differentially expressed genes (DEGs) were defined with adjusted p‐value (q) <0.05 and $|log_{2}\left(\mathrm{FC}\right)|\geq 0.25$.

### 4.10 Molecular Docking Simulations

3D structure of CYP2C9 complexed with a drug Lorsatan was retrieved from Protein Data Bank (PDB ID: 8VX0) [54]. The top bioactive molecules (CHEBL1466628, CHEMBL1353420, and CHEMBL1742011) were prioritized from our classification model. Top ten drugs or metabolites of AD genetics‐informed FPR1 and AD multi‐omics‐informed ADA2A were inspected. 3D structures of FPR1 or ADA2A were retrieved from AlphaFold2 database [86]. The preparation of 2D structures of small molecules and 3D structures of proteins refer to our previous study [59]. Molecular docking was processed by AutoDock Vina (version 1.1.2) [87]. Heme was kept for CYP2C9 molecular docking. The top ten binding modes were investigated, and the best binding poses were selected for further analysis. Detailed binding analysis were conducted by using PyMOL (version 3.0.3).

### 4.11 Statistical Analysis

The performance metrics for our benchmark results are presented by the mean and the 95% confidence internal of the standard error of the mean. The sample size (the number independent trials starting from different random seeds) was chosen to be five. $\chi^{2}$ p‐value tests were performed for select analysis. The genetics and multi‐omics statistical analysis is described in Section 4.9. Analysis was performed using python scripts and the NumPy and Scikit‐learn libraries.

## Conflicts of Interest

The authors declare no conflicts of interest.

## Supporting information




**Supporting File**: advs73607‐sup‐0001‐SuppMat.pdf.

## Data Availability

Our Multi‐View code and models are available on GitHub (https://github.com/BiomedSciAI/biomed‐multi‐view) and Hugging Face (https://Huggingface.co/ibm‐research/biomed.sm.mv‐te‐84m).

## APPENDIX A Pre‐Trained Models Learn Comprehensive Embeddings

Image, Graph, and Text offer intrinsically different views of chemical structure. However, it is expected that there is at least some overlap in their description of the embedding space. We sample a subset of 10,0000 molecules from the pre‐training set to perform the following analysis. In order to characterize the pre‐trained embeddings, we compute the correlation between their Euclidean distances in Figure S3A. It can be seen that of the three single‐view embeddings, Image is the most distinct. Text and Graph are highly correlated to each other (c=0.7), indicating that these two representations have a comparable description of molecular similarity.

Pre‐trained molecular embeddings should ideally capture important aspects of the chemical structures of the underlying molecules. While there is no absolute way of measuring this, comparison with fingerprints that have been developed by domain experts can serve as a sanity check on whether pre‐trained embeddings are in line with chemical knowledge [12]. Four common fingerprint descriptors (Morgan, atom pair, MACCS, and topological torsion) are chosen for this purpose [88, 89, 90, 91]. We first compute the correlation between fingerprints, and while the atom pair fingerprint is most correlated with the others, overall the chosen descriptors are moderately correlated and yield different descriptions of the molecules (see Figure S3A).

The correlations are computed between the Euclidean distance of the pre‐trained models and Tanimoto distance of the fingerprints and plotted in Figure S3B. It can be seen that there is moderate correlation between pre‐trained embedding and the fingerprints, demonstrating the validity of the embeddings. Overall, Text exhibits the highest correlation, followed by Graph and Image. Image is most correlated to the MACCS fingerprint, which is expected given that it is used in one of the ImageMol model's pre‐training tasks [21]. Finally, we note that the correlation with fingerprints only serves as a sanity check and strong agreement with fingerprints is not necessarily desirable for all down stream property prediction tasks, because each fingerprint is tailored using a limited set of hand‐engineered features. A key objective of learning pre‐trained multi‐view embeddings is to achieve richer, more flexible representations.

## APPENDIX B Single‐ and Multi‐ View Performance on Downstream Tasks

Here, we focus on comparisons of single view Graph, Text, and Image models and the multi‐view model in terms of their performance. All are fine‐tuned in the same code base with hyper‐parameter optimization (primarily the learning rate, see Section 4.8). The area under the receiver‐operator curve (ROC‐AUC) is the chosen metric for the classification tasks and root mean square error (RMSE) or mean absolute error (MAE) is chosen for regression [36]. Figure S4A and Tables S4–S8 report the numerical values for experiments with 95% confidence intervals. Regression metrics are scaled in so as to be plotted alongside ROC‐AUCs (see Section 4.8).

We find that overall that the Graph model outperforms Image and Text on our benchmark tests. The Graph model performs marginally worse than other single‐views in only a handful of examples (e.g., BBBP, QM7). We find that the multi‐view model performs robustly across all tasks, spanning areas such as molecule solubility prediction (ESOL, Lipophilicity), binding activity assay‐based classification (HIV, MUV) and ADME‐Tox based tasks. By ‘robustness’ we indicate that the multi‐view mode yields similar performance to the highest‐ranked single view and does not produce a poor result on any tested dataset (see Figure S4A).

In the multi‐view aggregator, the $m^{\text{th}}$ view to the overall embedding vector, $z_{i}^{\text{mv}}$, is weighted by coefficient $\alpha^{m}$,

(3)
$$\begin{array}{cc} z_{i}^{\text{mv}} & \propto \sum_{m\in M}\alpha^{m}z_{i}^{m} \end{array}$$

Therefore, an advantage of the multi‐view late‐fusion model architecture is that the contributions of single views to each task can be readily recovered through the aggregator weights. The α values are visualized as a heat map for MoleculeNet tasks in results in Figure S4B. We note that the pre‐trained aggregator (see Section 4.5) is free to evolve during fine‐tuning and adapt to the downstream tasks. The Graph model is heavily weighted for most tasks, which is consistent with the observation that Multi‐View performance is generally similar to Graph. A complementary analysis measuring the agreement between different views of selected fine‐tuned tasks is described in Section 4.7 and shown in Figures S5 and S6. For CYP models, both Graph and Image are nearly equally correlated with the multi‐view model, and this degeneracy can be related to trends in Figure S4B.

With the exception of QM7, the Text Model is a minor contributor to the multi‐view fine‐tuned embeddings and while the predictions between text and multi‐view are correlated, they exhibits the weakest relation (see Figures S5 and S6). This could be due to it being similar to Graph and yet the Graph model is a stronger single view model. This observation requires further study and could be related to previous results shown for multi‐modal architectures [24]. Overall, it can be stated that Graph and Image represent more distinct embeddings (see Section A) and are more complementary.
