# Supplementary material for: Multi‐View Biomedical Foundation Models for Molecule‐Target and Property Prediction
Source: Adv Sci (Weinh). 2026 Jan 28;13(14):e17840. doi: 10.1002/advs.202517840 (PMC12970179; doi:10.1002/advs.202517840)
Supplement: Supplementary file 1 — Supporting File: advs73607‐sup‐0001‐SuppMat.pdf. [file ADVS-13-e17840-s001.pdf]

SUPPORTING INFORMATION

Multi-view biomedical foundation models for molecule-target and property prediction

Parthasarathy Suryanarayanan[1*], Yunguang Qiu[2,3*], Shreyans Sethi[4], Diwakar Mahajan[1], Hongyang Li[1], Yuxin Yang[2], Elif Eyigoz[1], Aldo Guzmán-Sáenz[1], Daniel E. Platt[1], Timothy H. Rumbell[1], Kenney Ng[5], Sanjoy Dey[1], Myson Burch[1], Bum Chul Kwon[5], Pablo Meyer[1], Feixiong Cheng[2,3,6], Jianying Hu[1], Joseph A. Morrone[1]

[1]IBM TJ Watson Research Center, 1101 Kitchawan Rd., Yorktown Heights, NY, 10598, USA

[2]Cleveland Clinic Genome Center, Lerner Research Institute, Cleveland Clinic, Cleveland, OH, 44195, USA

[3]Genomic Medicine Institute, Lerner Research Institute, Cleveland Clinic, Cleveland, OH, 44195, USA

[4]IBM Research - Almaden, 650 Harry Rd, San Jose, CA, 95120, USA

[5]IBM Research, 314 Main St, Cambridge, MA 02142, USA

[6]Department of Molecular Medicine, Cleveland Clinic Lerner College of Medicine, Case Western Reserve University, Cleveland, OH, 44195, USA

Correspondence
Joseph A. Morrone, IBM TJ Watson Research Center, 1101 Kitchawan Rd., Yorktown Heights, NY, 10598, USA
Email: jamorron@us.ibm.com

This file contains supporting figures and tables for 'Multi-view biomedical foundation models for molecule-target and property prediction.' Figures S1-S8 and Tables S1-S9 are included.

[*]These authors contributed equally to this work.

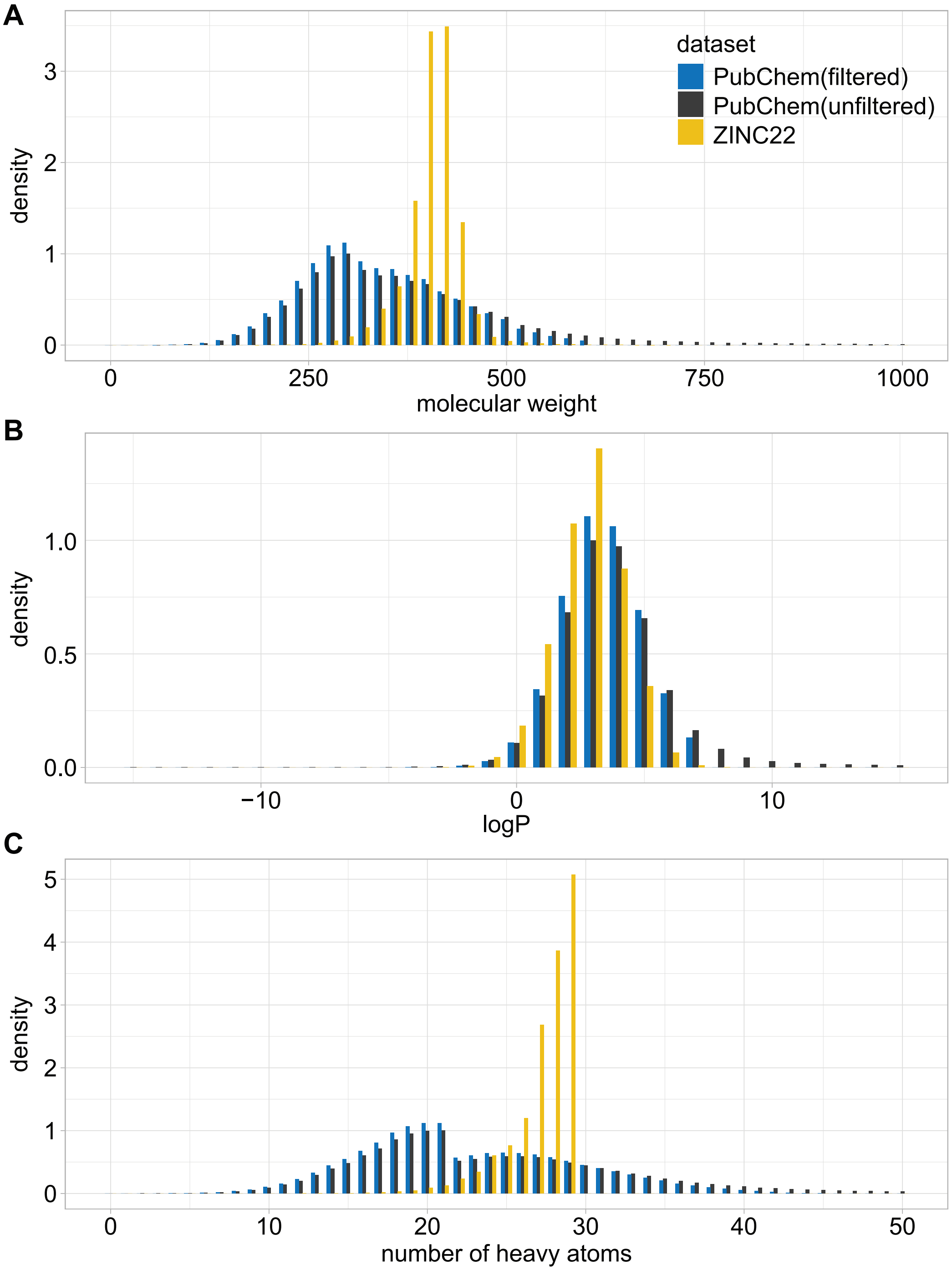

FIG. S1 Molecular properties of the pre-training dataset. Histograms of molecular weight (A), logP (B), and number of heavy atoms (C) are shown. Distributions are plotted for the filtered PubChem set used to build our foundation models (light blue), the full PubChem set (dark blue) and the molecules of ZINC22 that comprise the remainder of our dataset (yellow).

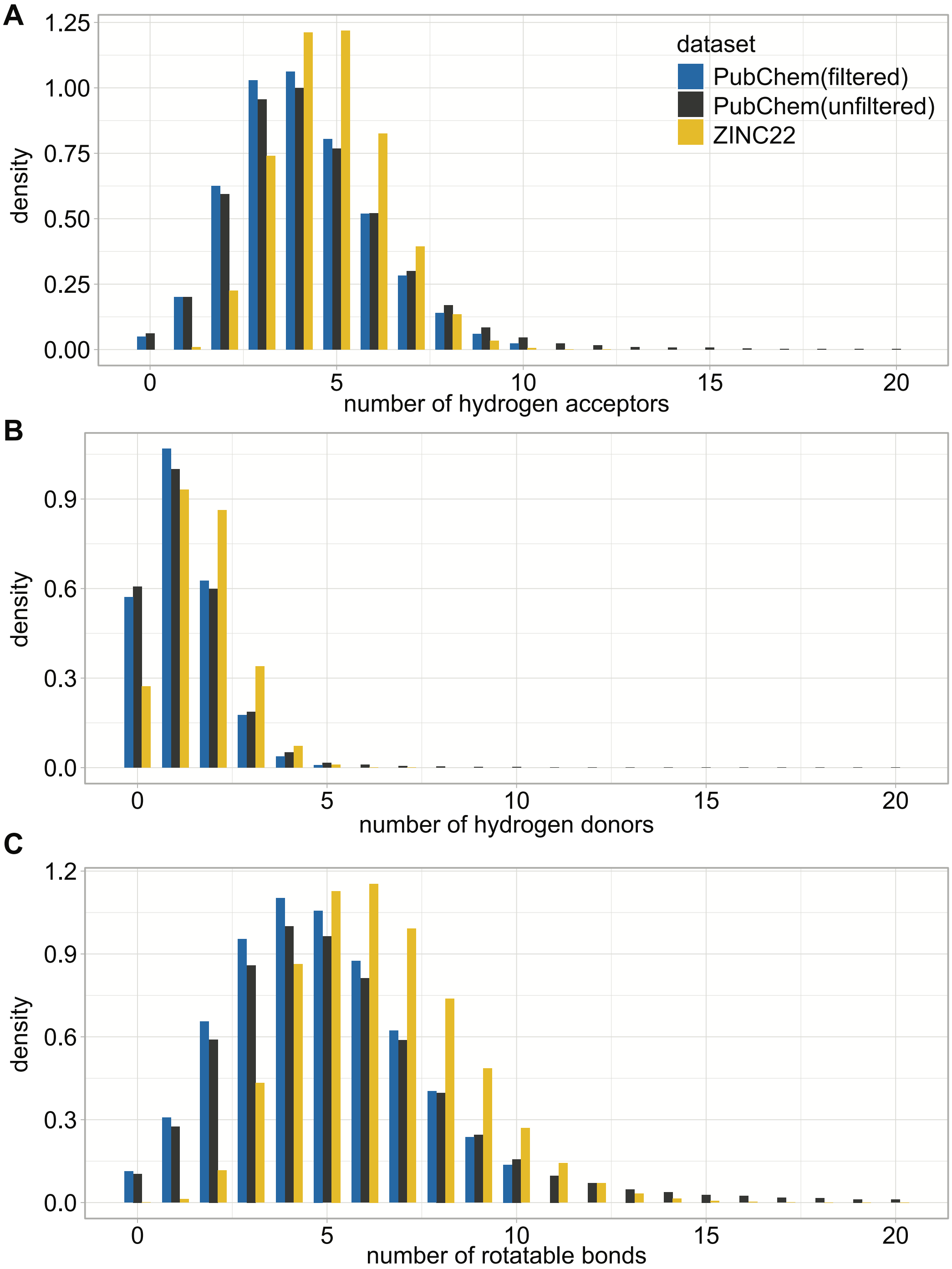

FIG. S2 Molecular properties of the pre-training dataset. Number of hydrogen bond acceptors (A), number of hydrogen bond donors (B), and number of rotatable bonds (C) are shown. Distributions are plotted for the filtered PubChem set used to build our foundation models (light blue), the full PubChem set (dark blue) and the molecules of ZINC22 that comprise the remainder of our dataset (yellow).

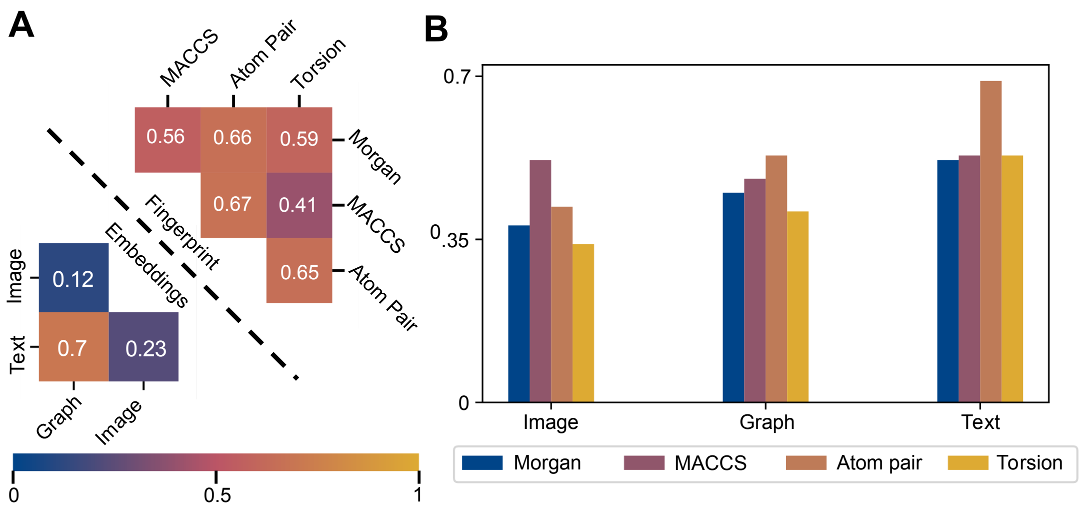

FIG. S3 (A) The elements of the correlation matrix between the three-single view embeddings (lower triangle) and between four fingerprints (upper triangle). (B) The correlation between four fingerprints and the pre-trained, Image (left grouping), Graph (center grouping) and Text (right grouping) embeddings.

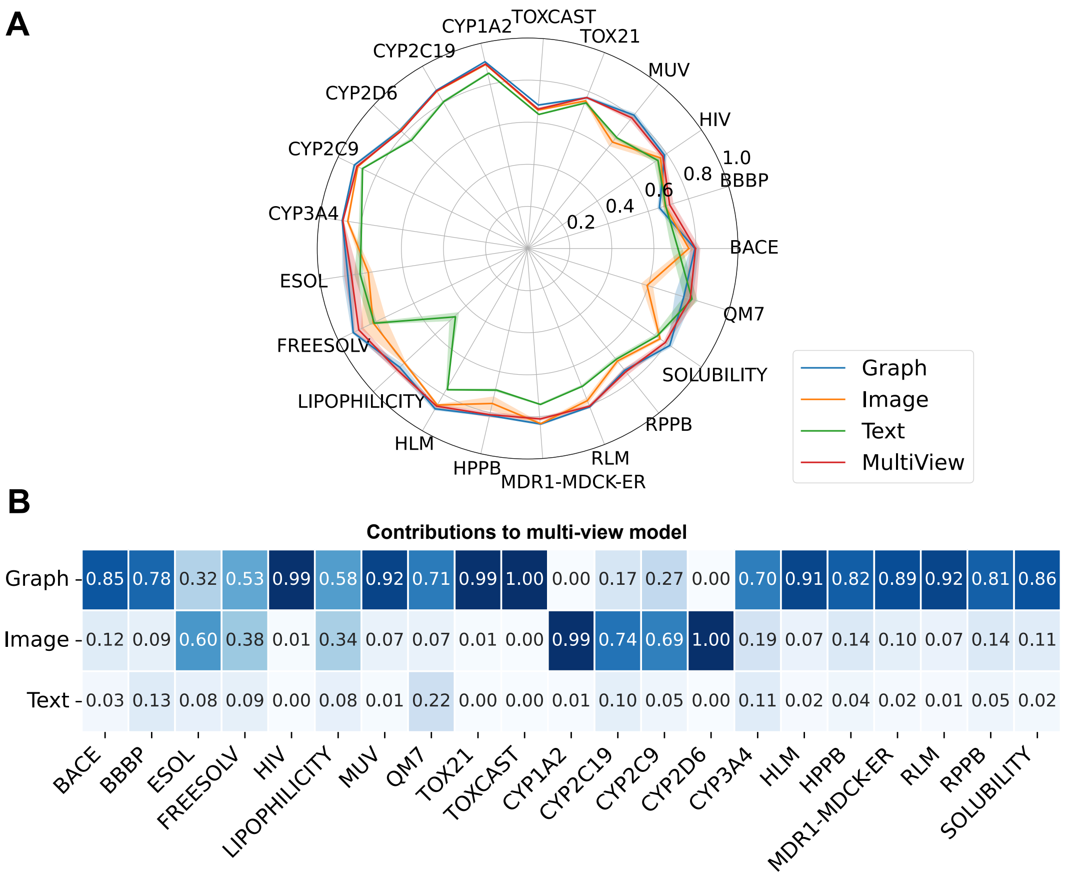

FIG. S4 (A) Performance of Graph (blue line), Image (orange line), Text (green line) and multi-view model (red line) fine-tuned on diverse downstream tasks. Train, validation, and test splitting is performed as described in the text. Classification tasks are characterized by ROC-AUC. Regression metrics (RMSE, MAE) are scaled so that they can be plotted with ROC-AUC values. The 95% confidence internal is depicted by the shaded region around each line and is based on fine-tuning for each task with 5 trials initialized from different random seeds. (B) Heat map of the weights α that the multi-view model assigns to Graph, Text and Image model for a subset of tasks shown in panel A.

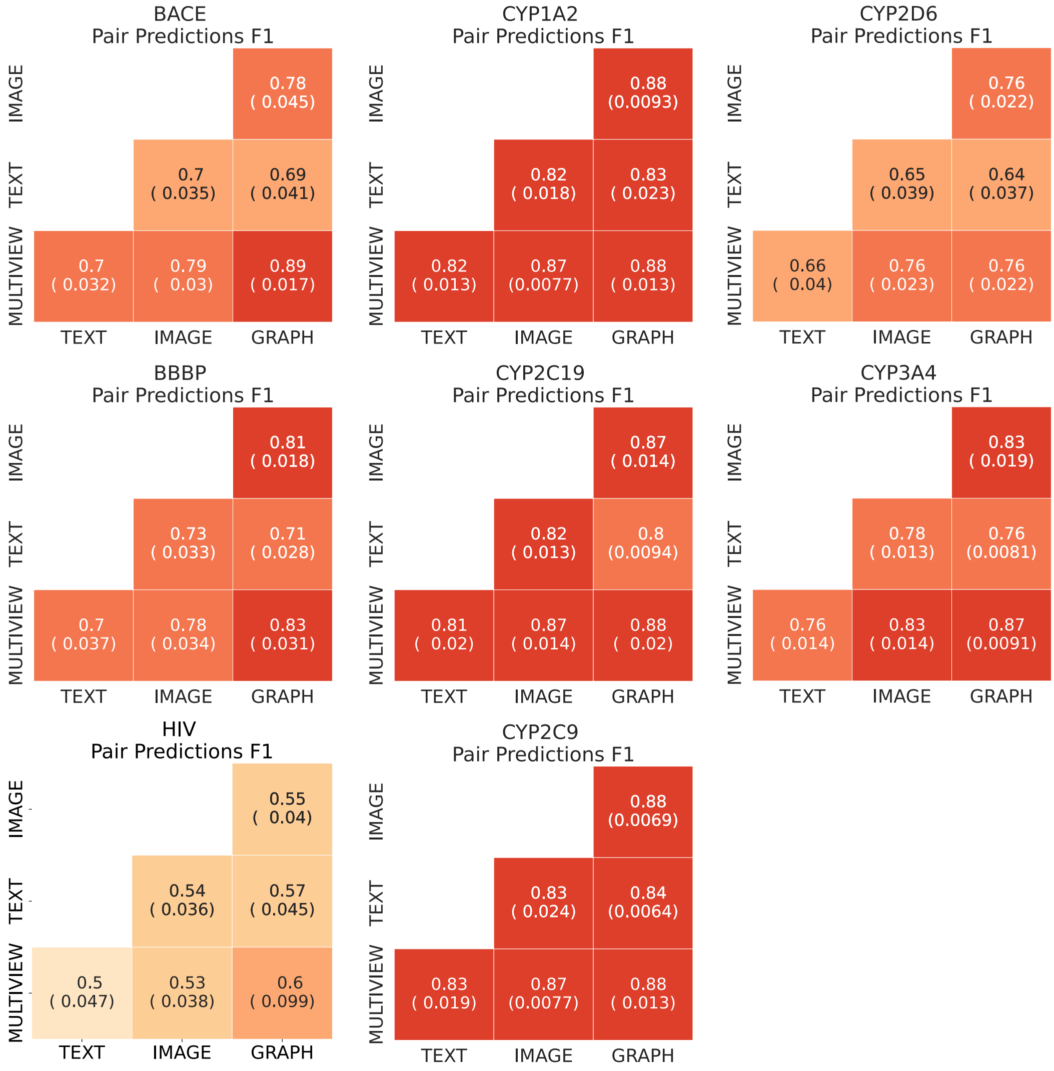

FIG. S5 $F1$ scores are shown between pairs of single-view and multi-view models. Results are shown for single task classification datasets. Higher F1 values indicate more agreement between the models vs. disagreements. In the case of CYP models the agreement between multi-view - Image, multi-view Graph and Image-Graph is nearly the same, indicating that the models yield largely the same outcomes. For other models, the Graph - multi-view yields the highest $F1$ score as expected from Fig. 2C in the main text. The p-value for each measurement is given in parenthesis.

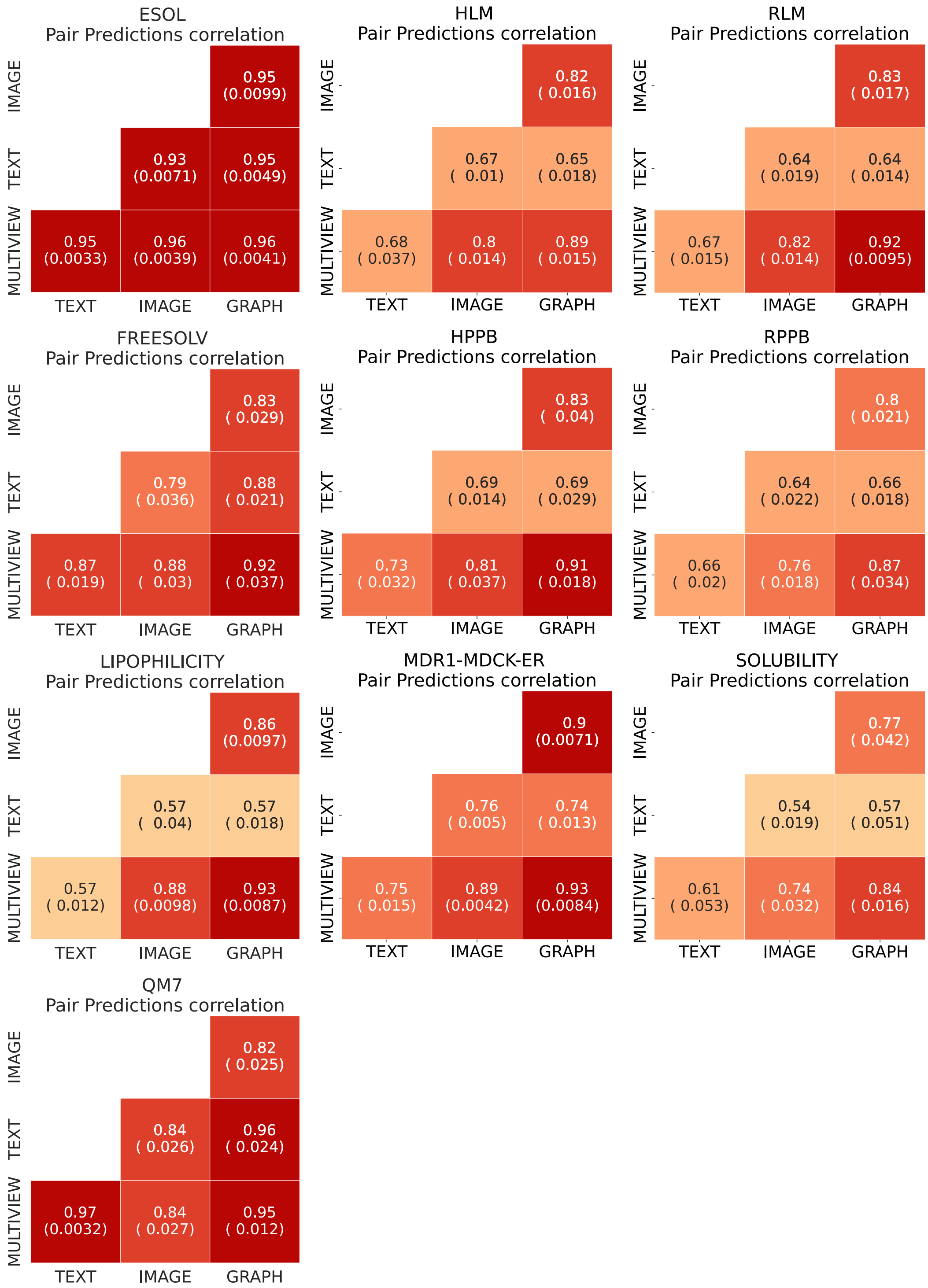

FIG. S6 Pearson corelation scores are shown between pairs of models for regression tasks. A higher number indicates greater corelation in the model predictions. For ComputationalADME tasks, Graph is the most correlated to multi-view as expected from Fig. 2C in the main text. In the case of QM7 the correlation between Text and multi-view is relatively high compared with other datasets. The p-value for each measurement is given in parenthesis.

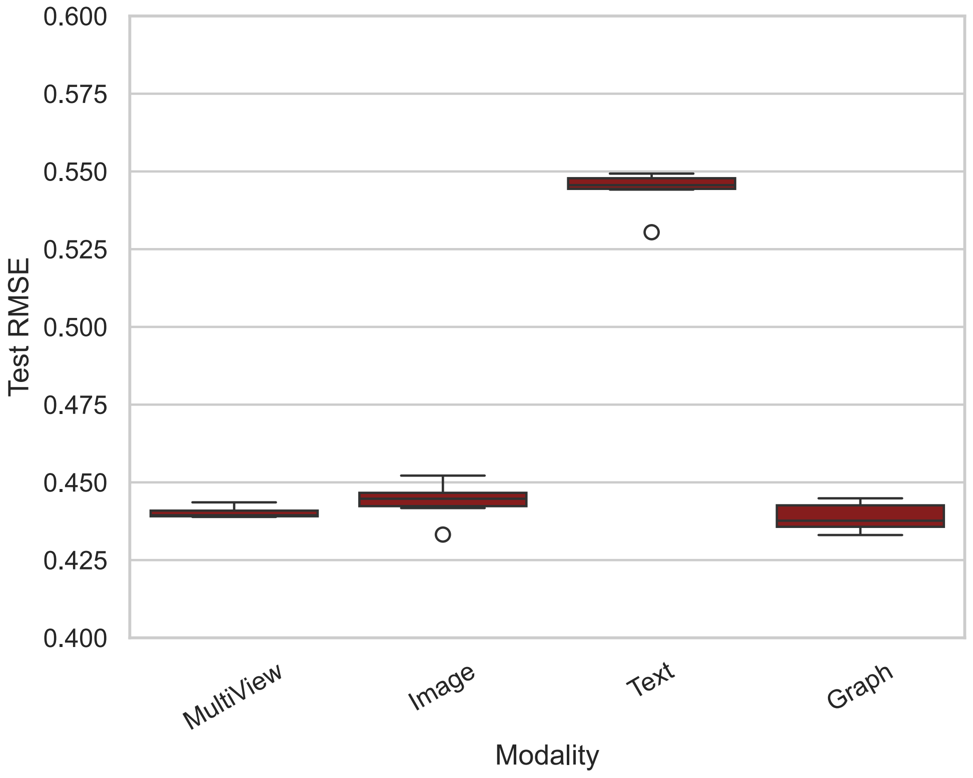

FIG. S7 RMSE for multi-view, Image, Graph, Text model fine-tuned on the Davis dataset. The Davis dataset contains the interaction of 72 kinase inhibitors with 442 kinases. Results are compared with reference value of Gorantla, et al. which is ≈ 0.48

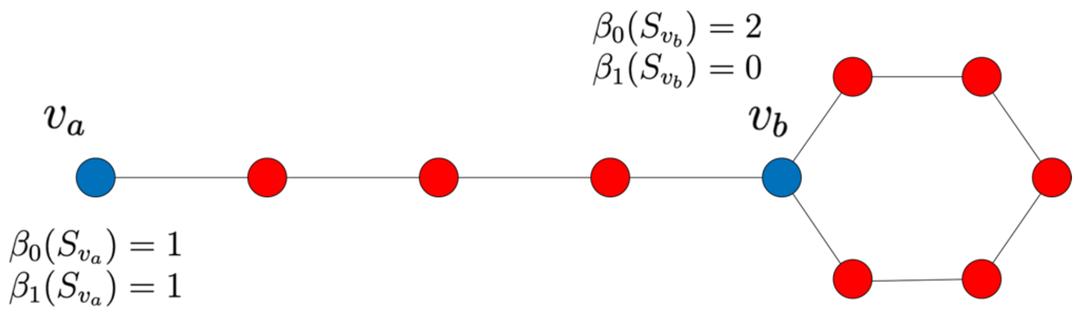

FIG. S8 Graph topology depiction that illustrates Betti numbers at a given node. Refer to the text for more details.

Model	Views	Encoders	Fusion / Training Paradigm
**MolPROP**	SMILES + 2D Graph	ChemBERTa-2 pretrained with MLM+MTR; GCN/GATv2 for graph encoding	Heavy-atom SMILES token embeddings mapped to graph nodes and concatenated; fine-tuned on seven regression/classification tasks (lipophilicity, solubility, solvation energy, atomization energy, toxicity, permeability, BACE).
**MoleSG**	SMILES + 2D Graph	SMILES and graph encoders feeding a shared Transformer backbone	Non-overlapping masking on each modality; joint reconstruction of SMILES and graph structure; pretrained on large unlabeled data and fine-tuned on 14 downstream tasks.
**MMFDL**	SMILES + Fingerprints + 2D Graph	Transformer-Encoder (SMILES); BiGRU with attention (fingerprints); two-layer GCN (graph)	Late fusion using one of five methods (LASSO, ElasticNet, RF, GB, SGD); Tri_SGD performs best; supervised training on six ADMET/physchem datasets and PDBbind.
**DLF-MFF**	FP + 2D Graph + 3D Structure + 2D Image	MLP (fingerprints); GCN (2D graph); geometric GNN (3D); CNN (image)	Late concatenation of four modality-specific representations followed by fully connected layers; trained on ESOL, FreeSolv, and Lipophilicity and applied to SARS-CoV-2 antiviral screening.
**PremuNet**	SMILES / FP + 2D Graph + 3D Structure	Transformer (1D); GAT–GCN hybrid (2D); geometric message passing (3D); PremuNet-L/H variants	Two-stage training: modality-specific pretraining, then Fusion-Net with concatenation and cross-modal attention; evaluated on eight MoleculeNet datasets.
**ISMol**	2D Image + SMILES	Vision Transformer (image); Transformer chemical language model (SMILES)	Bidirectional cross-attention aligns image and SMILES embeddings; supervised learning on 14 ADMET-related datasets.
**CGIP**	2D Graph + 2D Image	14-layer DeeperGCN (graph); ResNet-18 (image)	Multimodal contrastive pretraining with intra-modal (image–image, graph–graph) and inter-modal (graph–image) losses; pretrained on ∼10M PubChem molecules and fine-tunedon 12 benchmarks (BBBP, Tox21, ToxCast, SIDER, ClinTox, BACE, Estrogen, MetStab, FreeSolv, ESOL, Lipo, QM7).
**FP-GNN**	Fingerprints + 2D Graph	ANN over concatenated MACCS, PubChem, and Pharmacophore ErG fingerprints; multi-layer GAT (graph)	Late fusion using concatenation and fully connected layers; evaluated on 13 public datasets, LIT-PCBA, and 14 phenotypic breast-cancer screens; shows robust performance and balanced fingerprint/graph contributions.

TABLE S1 Representative multi-view molecular representation learning methods, summarized by view, encoder design, and fusion strategy.

dataset<br>model	ESOL	FREESOLV	LIPOPHILICITY	QM7
MultiView	0.853(35)	2.046(121)	0.654(12)	74.1(29)
Unprojected Gating	0.961(38)	2.192(51)	0.694(16)	83.8(59)
Projected Gating	0.939(14)	2.251(62)	0.693(9)	73.8(24)
Projected Gating with Feature	0.953(17)	2.225(6)	0.687(14)	72.7(50)

TABLE S2 Regression metrics (RMSE/MAE) for default multi-view model compared against other late fusion approaches that were explored (see Sec. 4.5). 95% confidence internal is based on fine-tuning for each task with 5 trials initialized from different random seeds and is given in parenthesis.

dataset<br>model	BACE	BBBP	HIV	MUV
MultiView	0.798(23)	0.706(19)	0.778(11)	0.794(14)
Unprojected Gating	0.798(27)	0.708(18)	0.754(8)	0.740(19)
Projected Gating	0.784(8)	0.690(8)	0.761(7)	0.734(18)
Projected Gating with Feature	0.789(9)	0.707(7)	0.757(4)	0.734(11)

TABLE S3 Classification metrics (ROC-AUC) for default multi-view model compared against other late fusion approaches that were explored (see Sec. 4.5). 95% confidence internal is based on fine-tuning for each task with 5 trials initialized from different random seeds and is given in parenthesis.

Task	Group	Description	$N_{samples}$	N_{tasks}	type	metric	split
BACE	MoleculeNet	Inhibition of human beta secretase 1	1513	1	C	roc-auc	size-ordered scaffold
BBBP	MoleculeNet	Blood brain barrier penetration	2039	1	C	roc-auc	size-ordered scaffold
HIV	MoleculeNet	Inhibition of HIV viral replication	41127	1	C	roc-auc	size-ordered scaffold
MUV	MoleculeNet	PubChem activities	93087	17	C	roc-auc	size-ordered scaffold
TOX21	MoleculeNet	Toxicity data	7831	12	C	roc-auc	size-ordered scaffold
TOXCAST	MoleculeNet	Toxicity data	8576	617	C	roc-auc	size-ordered scaffold
ESOL	MoleculeNet	Water solubility data for organics	1128	1	R	rmse	size-ordered scaffold
FREESOLV	MoleculeNet	Hydration free energy	642	1	R	rmse	size-ordered scaffold
LIPOPHILICITY	MoleculeNet	Octanol/water distribution coeff.	4200	1	R	rmse	size-ordered scaffold
QM7	MoleculeNet	Electronic properties from DFT	6830	1	R	mae	size-ordered scaffold
CYP1A2	CYP	Inhibition of CYP1A2 isoform	11725	1	C	roc-auc	balanced scaffold
CYP2C19	CYP	Inhibition of CYP2C19 isoform	11533	1	C	roc-auc	balanced scaffold
CYP2C9	CYP	Inhibition of CYP2C9 isoform	11722	1	C	roc-auc	balanced scaffold
CYP2D6	CYP	Inhibition of CYP2D6 isoform	11540	1	C	roc-auc	balanced scaffold
CYP3A4	CYP	Inhibition of CYP3A4 isoform	11118	1	C	roc-auc	balanced scaffold
HLM	ComputationalADME	Human liver microsomal stability	3087	1	R	rmse	random
HPPB	ComputationalADME	Human plasma protein binding	1801	1	R	rmse	random
MDR1-MDCK-ER	ComputationalADME	MDR1-MDCK efflux ratio	2642	1	R	rmse	random
RLM	ComputationalADME	Rat liver microsomal stability	3054	1	R	rmse	random
RPPB	ComputationalADME	Rat plasma protein binding	879	1	R	rmse	random
SOLUBILITY	ComputationalADME	Water solubility data	2173	1	R	rmse	random

TABLE S4 Description of benchmark tasks that are plotted in Fig. 2 in the main text, including CYP inhibition, ComputationalADME, and selections from MoleculeNet. Task types are classification (C) or regression (R).

dataset	ESOL	FREESOLV	LIPOPHILICITY	QM7
model				
GCN	1.350(54)	2.575(22)	0.818(15)	102.185(7768)
ChemBERTa-77M-MLM	1.049(31)	2.260(46)	0.763(16)	72.270(1059)
ChemBERTa-77M-MTR	0.873(23)	3.186(105)	0.696(8)	72.860(1509)
Graph	0.836(26)	1.900(36)	0.671(21)	77.324(4371)
Image	0.934(2)	2.426(322)	0.701(8)	95.584(333)
Text	0.889(22)	2.422(9)	1.024(32)	73.858(3463)
MultiView	0.853(35)	2.046(121)	0.654(12)	74.105(2883)

TABLE S5 MoleculeNet Size-ordered scaffold results for MoleculeNet regression tasks with 95% confidence interval in parenthesis. Confidence internal is based on fine-tuning for each task with 5 trials initialized from different random seeds. Metric is MAE for QM7, RMSE for other tasks.

dataset	BACE	BBBP	HIV	MUV	TOX21	TOXCAST
model						
GCN	0.733(7)	0.676(28)	0.735(1)	0.666(24)	0.730(8)	0.628(8)
ChemBERTa-77M-MLM	0.779(9)	0.722(5)	0.756(8)			
ChemBERTa-77M-MTR	0.736(130)	0.683(7)	0.741(7)			
Graph	0.795(21)	0.656(11)	0.787(14)	0.810(12)	0.770(8)	0.683(4)
Image	0.766(25)	0.684(1)	0.764(23)	0.647(25)	0.754(7)	0.659(5)
Text	0.709(27)	0.681(7)	0.743(15)	0.673(8)	0.744(7)	0.639(2)
MultiView	0.798(23)	0.706(19)	0.778(11)	0.794(14)	0.770(3)	0.664(8)

TABLE S6 Computed ROC-AUC for test set for size-order scaffold split for selected MoleculeNet classification tasks with 95% confidence interval in parenthesis. Confidence internal is based on fine-tuning for each task with 5 trials initialized from different random seeds.

dataset	CYP1A2	CYP2C19	CYP2C9	CYP2D6	CYP3A4
model					
GCN	0.869(2)	0.836(3)	0.891(4)	0.798(6)	0.832(6)
ChemBERTa-77M-MLM	0.891(2)	0.835(2)	0.888(1)	0.789(3)	0.866(2)
ChemBERTa-77M-MTR	0.898(3)	0.855(1)	0.902(2)	0.809(4)	0.881(2)
Graph	0.910(2)	0.867(4)	0.914(6)	0.827(7)	0.891(2)
Image	0.896(3)	0.862(4)	0.896(3)	0.824(2)	0.865(6)
Text	0.855(2)	0.807(3)	0.877(2)	0.759(6)	0.804(7)
MultiView	0.899(2)	0.864(5)	0.900(7)	0.820(3)	0.888(5)

TABLE S7 ROC-AUC for CYP inhibition tasks with 95% confidence interval in parenthesis. Confidence internal is based on fine-tuning for each task with 5 trials initialized from different random seeds.

dataset<br>model	HLM	HPPB	MDR1-MDCK-ER	RLM	RPPB	SOLUBILITY
GCN	0.526(18)	0.557(12)	0.607(15)	0.565(7)	0.603(15)	0.539(18)
Graph	0.421(9)	0.486(5)	0.464(7)	0.492(6)	0.560(1)	0.482(12)
ChemBERTa-77M-MLM	0.494(5)	0.601(11)	0.560(5)	0.580(6)	0.617(6)	0.540(9)
ChemBERTa-77M-MTR	0.463(1)	0.501(9)	0.491(5)	0.532(6)	0.537(9)	0.506(7)
Image	0.441(6)	0.545(34)	0.465(6)	0.524(14)	0.616(11)	0.538(9)
Text	0.526(4)	0.611(7)	0.558(4)	0.599(5)	0.630(7)	0.563(9)
MultiView	0.435(1)	0.490(13)	0.488(9)	0.495(9)	0.551(18)	0.508(1)

TABLE S8 ComputationalADME random splitting results with 95% confidence interval in parenthesis. For these regression tasks RMSE is the chosen metric. Confidence internal is based on fine-tuning for each task with 5 trials initialized from different random seeds.

Category	AD-related GPCRs
Genetic evidence-supported	FPR1
Strong Multi-omics evidence-supported (at least differential expressed in 5 datasets)	ADA2A
Weak multi-omics evidence-supported (differential expressed in less than 5 datasets)	N: 31<br>PE2R3, P2Y12, 5HT2A, HRH3, OPRK, EDNRA, ACM3, CCR2, 5HT1D, NPY1R, 5HT1A, GASR, ACM2, ADA1D, CNR1, PE2R4, MCHR1, ADRB1, ACM5, MTR1A, 5HT2C, 5HT1B, DRD1, CCR5, 5HT1F, 5HT7R, DRD5, 5HT5A, NPY2R, DRD2, NPY5R

TABLE S9 Genetics- or multi-omics-informed AD-related GPCRs
